# Prognostic immune markers identifying patients with severe COVID-19 who respond to tocilizumab

**DOI:** 10.3389/fimmu.2023.1123807

**Published:** 2023-05-05

**Authors:** Sara De Biasi, Marco Mattioli, Marianna Meschiari, Domenico Lo Tartaro, Annamaria Paolini, Rebecca Borella, Anita Neroni, Lucia Fidanza, Stefano Busani, Massimo Girardis, Francesca Coppi, Anna Vittoria Mattioli, Giovanni Guaraldi, Cristina Mussini, Andrea Cossarizza, Lara Gibellini

**Affiliations:** ^1^ Department of Medical and Surgical Sciences for Children and Adults, University of Modena and Reggio Emilia School of Medicine, Modena, Italy; ^2^ Infectious Diseases Clinics, Azienda Ospedaliera Universitaria (AOU) Policlinico and University of Modena and Reggio Emilia, Modena, Italy; ^3^ Department of Anesthesia and Intensive Care, Azienda Ospedaliera Universitaria (AOU) Policlinico and University of Modena and Reggio Emilia, Modena, Italy; ^4^ Department of Metabolic Sciences and Neurosciences, Azienda Ospedaliera Universitaria (AOU) Policlinico and University of Modena and Reggio Emilia, Modena, Italy; ^5^ National Institute for Cardiovascular Research, Bologna, Italy

**Keywords:** SARS-CoV-2, COVID-19, tocilizumab, cytokines, B cells, T cells

## Abstract

**Introduction:**

A growing number of evidences suggest that the combination of hyperinflammation, dysregulated T and B cell response and cytokine storm play a major role in the immunopathogenesis of severe COVID-19. IL-6 is one of the main pro-inflammatory cytokines and its levels are increased during SARS-CoV-2 infection. Several observational and randomized studies demonstrated that tocilizumab, an IL-6R blocker, improves survival in critically ill patients both in infectious disease and intensive care units. However, despite transforming the treatment options for COVID-19, IL-6R inhibition is still ineffective in a fraction of patients.

**Methods:**

In the present study, we investigated the impact of two doses of tocilizumab in patients with severe COVID-19 who responded or not to the treatment by analyzing a panel of cytokines, chemokines and other soluble factors, along with the composition of peripheral immune cells, paying a particular attention to T and B lymphocytes.

**Results:**

We observed that, in comparison with non-responders, those who responded to tocilizumab had different levels of several cytokines and different T and B cells proportions before starting therapy. Moreover, in these patients, tocilizumab was further able to modify the landscape of the aforementioned soluble molecules and cellular markers.

**Conclusions:**

We found that tocilizumab has pleiotropic effects and that clinical response to this drug remain heterogenous. Our data suggest that it is possible to identify patients who will respond to treatment and that the administration of tocilizumab is able to restore the immune balance through the re-establishment of different cell populations affected by SARS-COV-2 infection, highlighting the importance of temporal examination of the pathological features from the diagnosis.

## Introduction

Most SARS-CoV-2 infections are asymptomatic or pauci-symptomatic. However, a proportion of individuals develop a severe disease, characterized by a progressive respiratory failure after the onset of dyspnea and hypoxemia (1). It is well known that the combination of hyperinflammation, dysregulation in T and B cell response and cytokine storm are responsible for the development of severe and eventually fatal COVID-19 (2–4). Plasma levels of several inflammatory cytokines, such as interleukin (IL)-6, tumor necrosis factor (TNF), granulocyte-macrophage colony-stimulating factor (GM-CSF), and granulocyte colony-stimulating factor (G-CSF) are indeed increased after SARS-CoV-2 infection, in patients with the severe disease (3–6).

IL-6 is one of the main pro-inflammatory cytokines. It regulates several aspects of innate and adaptive immunity, including the differentiation of B lymphocytes, cytotoxic T cells, and macrophage/monocyte functions (7, 8). IL-6 exists both as a soluble and membrane-bound molecule, and is typically found in the blood of healthy individuals at very low concentrations (1-5 pg/mL) (9). Its levels increase during acute and/or chronic inflammation (10, 11). IL-6 binds to IL-6 receptor (IL-6R, or CD126) and glycoprotein 130 (gp130) to form a hexameric complex that transduces IL-6 signal through the Janus kinase (JAK) and signal transducer and activator of transcription (STAT) pathway (10). In the context of disease, IL-6 can have both local and systemic deleterious inflammatory effects. Indeed, the association between high IL-6 concentration (> 80 pg/mL) and respiratory failure and/or death has been confirmed in several studies and posed the rationale for the use of IL-6 blockers in the management of patients with severe COVID-19 (12, 13).

Tocilizumab is a humanized antibody that targets IL-6R and inhibits the binding of IL-6 to both soluble and membrane forms of the receptor. It interferes with IL-6 signaling and dampen inflammation. The single-cell RNA-seq analysis of peripheral blood mononuclear cells (PBMCs) isolated from two severe COVID-19 patients treated with tocilizumab, at the severe and remission disease stages, revealed that a monocyte-associated cytokine storm is present in patients at the severe stage and that tocilizumab can weaken this inflammatory response (14). Both case-control studies and randomized trials of tocilizumab in COVID-19 have demonstrated that it improves survival and reduces the chances of progressing to invasive mechanical ventilation in hospitalized COVID-19 patients with hypoxia and systemic inflammation (15, 16). Data from the REMAP-CAP and RECOVERY trials revealed that combining corticosteroids with tocilizumab yields cumulative benefits for mortality and morbidity in severe COVID-19 (15, 17). However, as already observed for other immunotherapeutic drugs in other diseases, including chronic inflammatory diseases and cancer, a proportion of patients did not derive benefits from tocilizumab treatment (18, 19).

In the present study, we aimed at identifying possible prognostic biomarkers related to the response to this drug. For this reason, we profiled the plasma levels of several cytokines, chemokines and other soluble factors in patients with severe COVID-19 who responded or not to tocilizumab, and we examined how tocilizumab impacted the composition of peripheral immune cells.

## Results

### Modulation of plasma cytokines and chemokines during tocilizumab treatment

The plasma profiling of 62 cytokines, chemokines and other soluble factors involved in several immune responses was first performed in 23 patients with severe COVID-19, responding or not to tocilizumab. The drug was given twice, 12 hours apart, by intravenous or subcutaneous route (see Methods for details). Plasma molecules were quantified at the baseline (T0, before the beginning of treatment), two days (T2) and seven days (T7) after the first dose of the drug. Most patients were sampled during the acute phase of the infection: 13 patients responded to therapy, whereas 10 patients did not. Demographic information, clinical data, blood parameters and arterial haemogas analysis, collected at T0, are reported in **Supplementary Table 1**.

**Figure 1:**
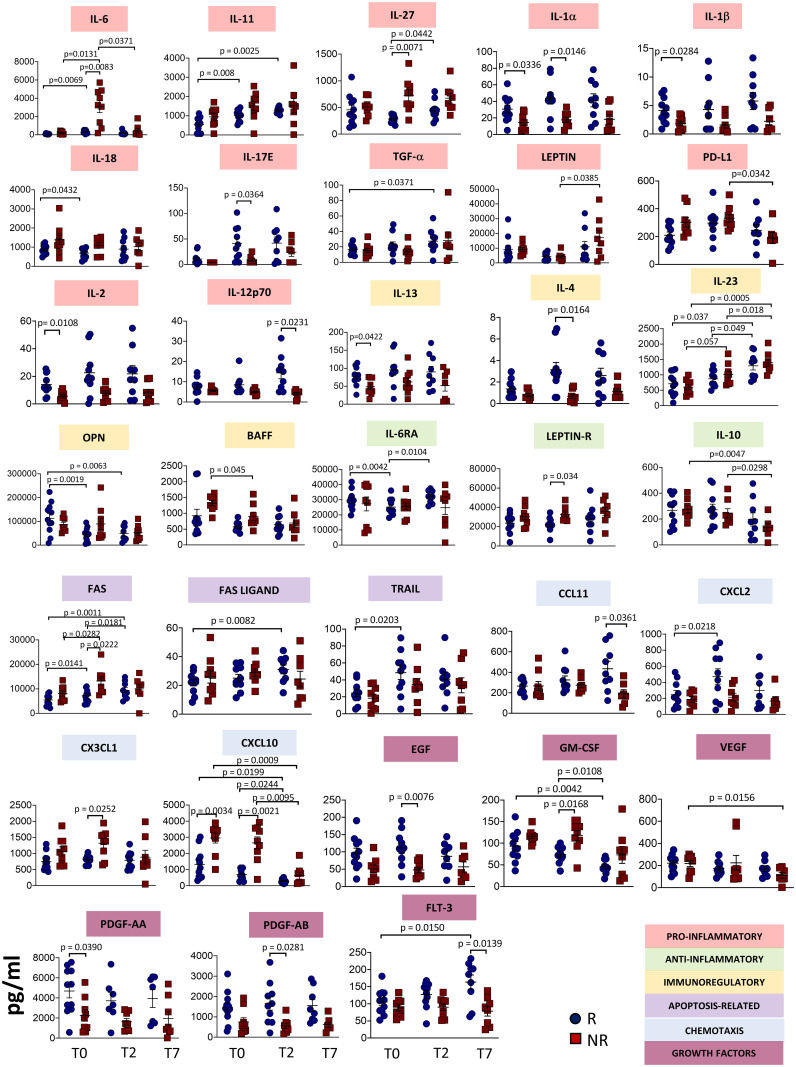
Plasmatic level of different soluble molecules in responder and non-responder patients. Quantification of cytokines and other mediators in plasma obtained from COVID-19 patients responding (R) or not (NR) to tocilizumab (n = 23). Data represent individual values, mean (centre bar) ± SEM (upper and lower bars). Statistical analysis by two-way ANOVA followed by Bonferroni correction; if not indicated, p-value is not significant.

**Figure 1** shows plasma levels of several cytokines/chemokines and other soluble factors in responder and non-responder patients. Concerning pro-inflammatory cytokines, we found that after 2 days of therapy IL-6 and IL-27 plasma concentrations strongly increased in non-responders. IL-11 levels did not differ between responders and non-responders, but increased in responders after therapy. Non-responders had lower basal levels of interleukin IL-1α, IL-1β, while in responders at T2 IL-18 decreased and IL-17E increased. Level of TGF-α increased over time in responders. Leptin level increased from T2 to T7 in non-responders, while PD-L1 decreased. IL-2 level was lower in non-responders at T0 and significant differences were observed at T7 between responders and non-responders for IL-12p70.

The concentrations of IL-6RA reached the minimum value at T2 in responders. At T2, Leptin receptor (Leptin R) was different between responders and non-responders, with higher level in the latter. IL-10 decreased during therapy in non-responders. IL-2 and IL-13 plasma levels were lower in non-responders at T0, while IL-4 level was lower at T2. Significant differences were observed at T7 between responders and non-responders for IL-12p70. We also noted that IL-23 increased after one week of treatment in both groups of patients. Levels of optineurin (OPN) decreased during therapy in patients responding to tocilizumab. The level of B-cell activating factor (BAFF) decreased in non-responders during therapy.

Apoptosis-related molecules have been investigated. FAS levels increased during therapy in responder and non-responder patients, but higher levels have been detected in non-responders at T2. FAS ligand and TRAIL levels increased in responders, at T7 and T2, respectively.

Plasma concentrations of C-C Motif Chemokine Ligand (CCL)-11 were lower in non-responders at T7, while C-X-C motif chemokine ligand (CXCL)-2 was higher at T2 in responders. CX3CL1 was higher at T2 in non-responders. CXCL10 plasma levels decreased over time in both groups of patients, but non-responders displayed higher levels at T0. This difference was maintained at T2 and at T7.

Regarding growth factors, in responders epidermal growth factor (EGF) was higher at T2 whereas GM-CSF decreased with time. Vascular endothelial growth factor (VEGF) decreased in non-responders over time. Levels of platelet-derived growth factor (PDGF)-AA were lower at the baseline in non-responders. (PDGF)-AB was lower in non-responders at T2. Fms related receptor tyrosine kinase 3 (FLT-3 ligand, also known as CD135) decreased after seven days in non-responders, but increased in responders. All the other soluble molecules remained unchanged in responders and non-responders (**Supplementary Figure 1**).

### Circulating plasmablasts decreased in responders after therapy

**Figure 2:**
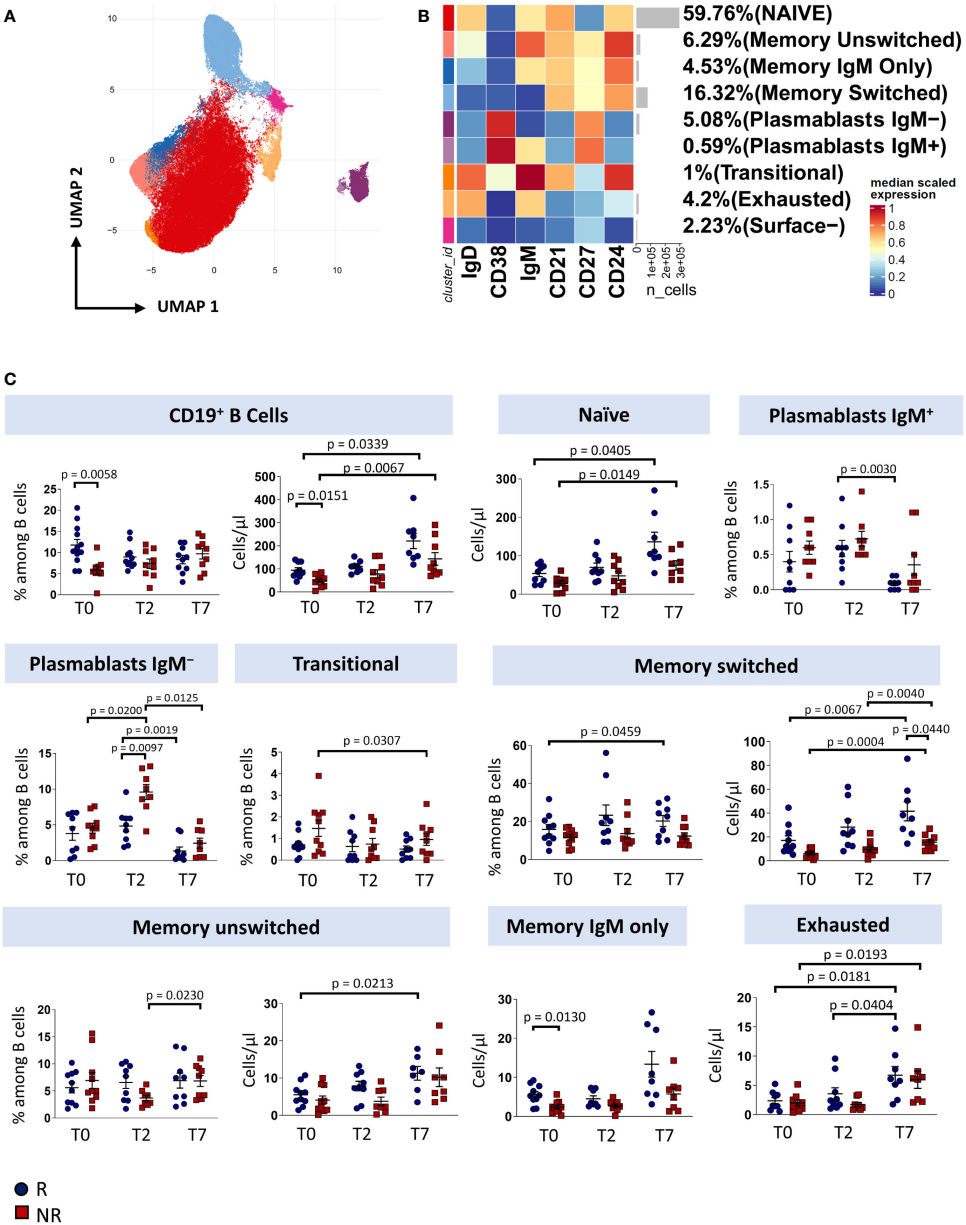
B cells landscape of COVID-19 patients treated with tocilizumab. **(A)** Uniform Manifold Approximation and Projection (UMAP) representation of the B-cell landscape. Each color is assigned according to the cluster identification palette. **(B)** Heatmap representing different B-cell clusters identified by FlowSOM, with relative identity and percentages. The colors in the heatmap represent the median of the arcsinh, 0-1 transformed marker expression calculated over cells from all the samples, varying from blue for lower expression to red for higher expression. Each cluster has a unique color assigned (bar on the left). **(C)** Statistical analysis of the different cell populations identified as in **(B)** Comparison between responders (R, blue circles) and non-responders (NR, red squares) at different treatment timepoints. The dot plots show the relative abundancies (left plot) and the absolute numbers (right plot) of populations found within B cells. Data represent individual percentage values (dots), median (center bar) and SEM (upper and lower bars). Statistical analysis by two-way ANOVA test with Bonferroni correction has been used. Exact p-value is indicated in the figure.

To investigate how immune responses vary in the presence of tocilizumab, we profiled B cells and T cells by using polychromatic flow cytometry. After unsupervised analysis, nine distinct cell clusters have been identified (UMAP and heatmap are reported in **Figures 2A, B**) on the basis of the surface protein expression (**Supplementary Figure 2**). Besides a population of cells negative for all the activation/differentiation markers analyzed, naïve B cells are defined as IgM^+^IgD^+^CD24^+^CD21^+^CD38^−^CD27^−^ while transitional B cells are IgM^+^IgD^+^CD24^+^CD21^+^CD38^+^CD27^−^ memory unswitched B cells are defined as IgM^+^IgD^+^CD24^+^CD21^+^CD38^−^CD27^+^ while memory switched B cells are IgM^−^IgD^−^CD24^+^CD21^+^CD38^−^CD27^+^. Memory IgM only B cells are IgM^+^IgD^-^CD24^+^CD21^+^CD38^−^CD27^+^. Plasmablasts are IgM^−/+^IgD^−^CD24^−^CD21^−^CD38^+^CD27^+^. Finally, exhausted B cells are those CD21^−^ and CD24^−^ (2).

Before therapy (T0), responders showed a higher percentage and absolute number of total B cells and a higher absolute number of memory IgM-only subpopulation compared to non-responders. After two days of therapy, plasmablasts not expressing IgM increases in non-responders compared to responder patients. Responders show an increase in both absolute number and percentage of memory switch B cell population compared to non-responders. After seven days of therapy, we observed an increase in absolute number of total B cells and naïve B cell subpopulation in both responder and non-responder patients, an increase in percentage of plasmablast IgM^+^ in responder patients, a decrease in percentage of plasmablast IgM^−^ and an increase in absolute number of exhausted B cell and surface- subpopulation in both groups. Finally, non-responder patients display a decrease of absolute number of transitional B cells (**Figure 2C**).

### Tocilizumab induces a redistribution in memory T cell pool

T cell compartment of patient with COVID-19 pneumonia displays marked T cell activation, senescence, exhaustion and skewing towards Th17 (3). Given that also T cells expressed CD126 and could be tuned by Tocilizumab administration, we investigated the T cells landscape of patients undergoing anti-IL6R-therapy.

**Figure 3:**
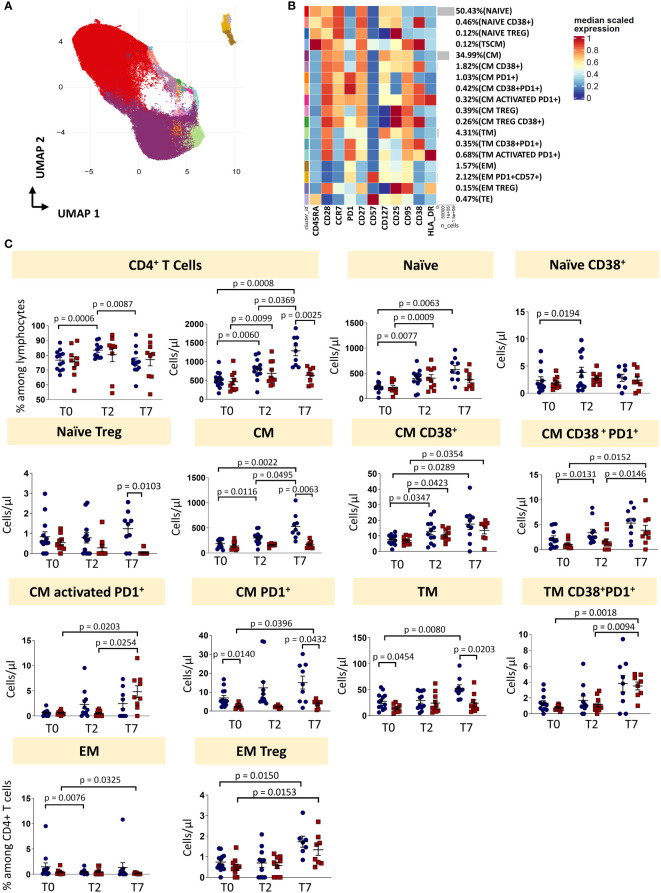
CD4 T cells landscape of COVID-19 patients treated with tocilizumab. **(A)** Uniform Manifold Approximation and Projection (UMAP) representation of the CD4 T cell landscape. Each color is assigned according to the cluster identification palette. **(B)** Heatmap representing different cell clusters identified by FlowSOM, with relative identity and percentages. The colors in the heatmap represent the median of the arcsinh, 0-1 transformed marker expression calculated over cells from all the samples, varying from blue for lower expression to red for higher expression. Each cluster has a unique color assigned (bar on the left). **(C)** Statistical analysis of the different CD4 T cells populations identified as in **(B)** Comparison between responders (R, blue circles) and non-responders (NR, red squares) at different treatment timepoints. The dot plots show the relative abundancies and/or absolute numbers of subpopulations found within CD4 T cells. Data represent individual percentage values (dots), median (center bar) and SEM (upper and lower bars). Statistical analysis by two-way ANOVA test with Bonferroni correction has been used. Exact p-value is indicated in the figure. T_SCM_, T cells stem memory; CM, central memory; TM, transitional memory; EM, effector memory; TREG, T regulatory cells; TE, terminally differentiated T cells re-expressing CD45RA.

Eighteen clusters of CD4^+^ T cells were recognized after unsupervised analysis on the basis of different expression of differentiation (CD45RA, CCR7, CD25, CD95, CD27, CD28, CD57) and activation markers (HLA-DR, CD28, PD1) (**Figures 3A, B**; **Supplementary Figure 3, 4**). Naïve T cells (N) were defined as expressing CD45RA, CCR7, CD27, CD28, CD127, CD25 and lack of expression of CD95, CD38 and HLA-DR. Moreover, a population of recently activated naïve T cells has been recognized as those expressing CD38. CD38 is a multifunctional molecule, belonging to the family of ectoenzymes and it induces intracellular calcium release. The expression of CD38 identifies a hypo-proliferative CD4^+^ T-cell subset that, following TCR stimulation, retains expression of naïve cell surface markers including CD45RA and CCR7 (20). We were able also to identify T cells stem memory (T_SCM_) as naïve cells expressing CD95 and CD38. Central memory T cells (CM) were those expressing CCR7, CD95 and not CD45RA, then four subsets of central memory were found: one expressing CD38, one expressing PD-1, one co-expressing CD38 and PD-1 and one activated (CD38^+^HLA-DR^+^) expressing also PD-1. Three populations of transitional memory (TM) were found as they do not express CD45RA and CCR7, but they express CD28. Moreover, one population of TM is phenotypically resting, one population is expressing CD38 and PD1 and another subset is highly activated as it expresses high level of HLA-DR, CD38 and PD-1. In particular, PD-1 is expressed during the early phase of T cell activation when naïve T cells differentiate into effector cells: it is rapidly expressed after antigen stimulation of naïve T cells with a kinetics of expression similar to the expression of early activation markers (CD69 and CD25) (21, 22). Effector memory T cells (EM) are characterized by the lack of the expression of CD28, CD45RA and CCR7. Two different populations of EM have been identified: one has been identified as a population of phenotypically resting cells and the other as exhausted cells as it expresses CD57 and PD-1. A population of terminally differentiated T cells (TE) were found as effector memory re-expressing CD45RA. Finally, putative T regulatory cells (T_reg_) were defined as those expressing CD25 and not expressing CD127. Four populations were found: naïve T_reg_ expressing CD45RA and CCR7, central memory T_reg_ as those not expressing CD45RA and expressing CCR7, central memory T_reg_ expressing also CD38 and effector memory T_reg_ as those characterized by the lack of expression of both previous markers.

The percentage of CD4^+^ T cells was influenced by therapy, as in responders it increased at T2 and decreased at T7. The absolute number increased at T2 and T7 in responders, and only from T0 and T2 in non-responders. At T7, the absolute number of CD4^+^ T cells was lower in non-responders if compared to responders. The absolute number of naïve T cells increased in responders (until T7) and non-responders (until T2). Naïve CD38^+^ T cells increased in responders from T0 to T2, while the absolute number of naïve Treg at T7 was higher in responders *vs* non-responders. Absolute number of CM T cells increased after treatment in responders, but not in non-responders. The same trend was observed for the absolute number of CM CD38^+^ T cells and CM CD38^+^ T cells expressing PD-1 for responders and non-responders. The absolute number of activated CM expressing PD-1 increased in non-responders, but did not change in responders. Absolute numbers of CM T cells expressing PD-1 were higher in responders at each time points. Absolute number of TM cells was higher at T0 and T7 in responders and increased during therapy for responder patients. The absolute number of TM expressing CD38 and PD-1 increased in non-responders after therapy, but did not change in responders. The percentage of EM T cells decreased in non-responders from T0 to T7 and in responders from T0 to T2. The absolute number of EM Treg increased in both responders and non-responders after therapy (from T0 to T7).

To sum up, even if the percentage of total CD4^+^ T is modulated by the therapy, the proportion of different CD4^+^ T cell subpopulations remained unchanged. However, the increased of the absolute number of CD4^+^ T cells in responders was likely responsible for all the differences found in the main subpopulations (**Figure 3C**, exact p-values are reported in the figures).

**Figure 4:**
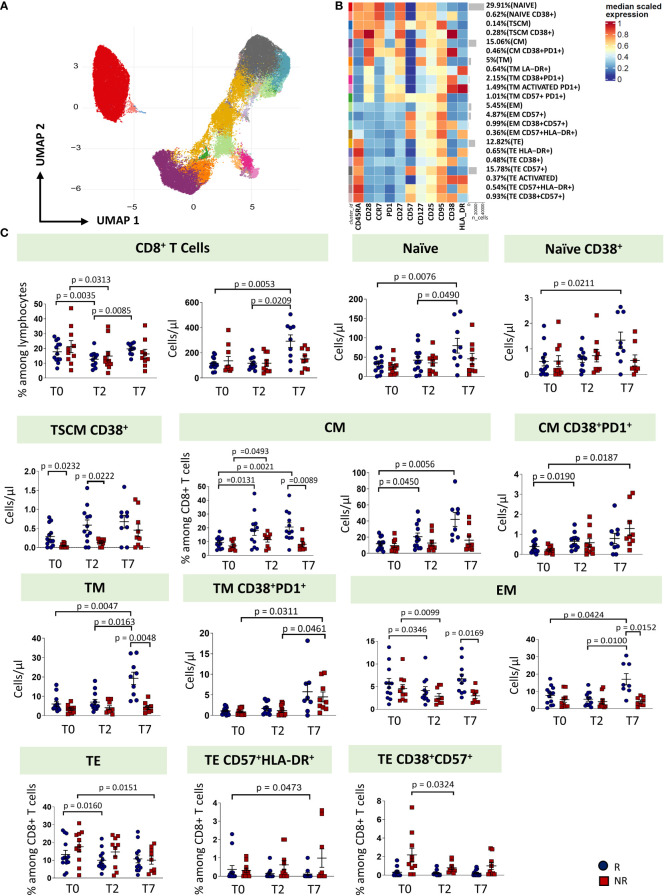
CD8 T cells landscape of COVID-19 patients treated with tocilizumab. **(A)** Uniform Manifold Approximation and Projection (UMAP) representation of the CD8 T cell landscape. Each color is assigned according to the cluster identification palette. **(B)** Heatmap representing different cell clusters identified by FlowSOM, with relative identity and percentages. The colors in the heatmap represent the median of the arcsinh, 0-1 transformed marker expression calculated over cells from all the samples, varying from blue for lower expression to red for higher expression. Each cluster has a unique color assigned (bar on the left). **(C)** Statistical analysis of the different CD8 T cells populations identified as in **(B)** Comparison between responders (R, blue circles) and non-responders (NR, red squares) at different treatment timepoints. The dot plots show the relative abundancies and/or absolute numbers of subpopulations found within CD8 T cells. Data represent individual percentage values (dots), median (center bar) and SEM (upper and lower bars). Statistical analysis by two-way ANOVA test with Bonferroni correction has been used. Exact p-value is indicated in the figure. T_SCM_, T cells stem memory; CM, central memory; TM, transitional memory; EM, effector memory; TE, terminally differentiated T cells re-expressing CD45RA.

Regarding CD8^+^ T cell subsets, twenty-two clusters of CD8 T cells have been identified by unsupervised analysis on the basis of different expression of differentiation (CD45RA, CCR7, CD25, CD95, CD27, CD28, CD57) and activation markers (HLA-DR, CD28, PD-1) (**Figures 4A, B**; **Supplementary Figures 5, 6**). These populations span the entire spectrum of differentiation and activation status. As for CD8^+^ T cell subsets, naïve cells, naïve cells expressing CD38, T_SCM_, T_SCM_ expressing CD38, CM, CM co-expressing CD38 and PD-1, TM, EM and TE were identified. Of the TM population, we found four different subsets: a subset of phenotypically resting cells, a subset of TM expressing HLA-DR, a subset of TM expressing CD38 and PD-1, a subset of activated TM also expressing PD1 and a subset of exhausted TM (CD57^+^PD1^+^). Moreover, we found four populations of EM: EM phenotypically resting, EM expressing only CD57, EM expressing CD38 and CD57, EM co-expressing CD57 and HLA-DR. Finally, seven populations of TE cells have been identified: TE phenotypically resting, TE expressing HLA-DR, TE expressing CD38^+^, TE expressing CD57, TE activated (CD38^+^HLA-DR^+^), TE co-expressing CD57 and HLA-DR, and TE co-expressing CD38 and CD57.

The percentage of CD8^+^ T cells decreased from T0 at T2 in both groups of patients, even if in responders the percentage increased at T7, reaching a similar level of T0. As far as the absolute number is concerned, responders were characterized by an increase of CD8^+^ T cells at T7. Similar absolute numbers of CD8^+^ T cells were observed in non-responders. At T7, responders were characterized by increased absolute number of naïve, naïve CD38^+^, CM, TM and EM T cells. The absolute number of T pedix SCM CD38^+^ was different at T0 and T2 between responders and non-responders, with higher level in responders. Non-responders had higher absolute number of CM CD38^+^PD-1^+^ and TM CD38^+^PD-1^+^, at T7. Moreover, at T7, responders showed higher levels of TM and EM if compared to non-responders. The percentages of TE decreased at T7 in non-responders and at T2 in responders. The percentage of TE CD38^+^CD57^+^ decreased at T2 in non-responders (**Figure 4C**, exact p values are reported in the figures).

**Figure 5:**
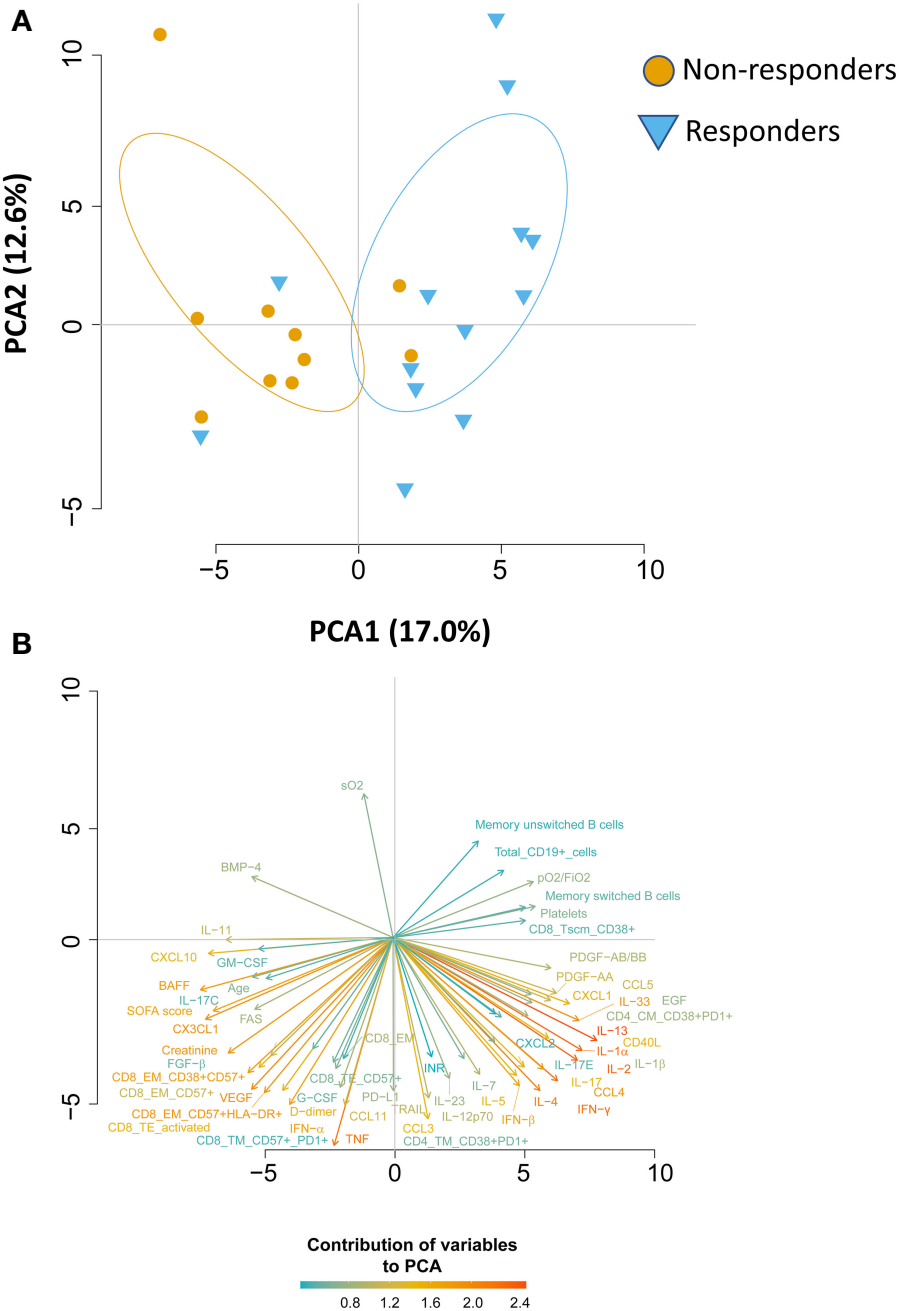
Principal Component Analysis (PCA) reveals that responder and non-responder patients are different before starting therapy. **(A)** PCA showing the distribution of responder and non-responder patients. **(B)** Plot displaying the variables as vectors, indicating the direction of each variable to the overall distribution. The strength of each variable is represented by colors: orange color represents a strong contribution, light blue color represents a milder contribution. Length and direction of the arrows indicate the weight and correlation for each parameter.

We wondered whether the two groups of patients could be identified based on immunological and clinical parameters before therapy initiation (T0), predicting who will benefit from therapy and who will not. By using the principal component analysis (PCA), we saw that non-responders clusterize on the left side of the first dimension (PC1, representing 17% of variance), while responder patients clusterize on the right side (**Figure 5A**), meaning that before starting therapy they were extremely different from both immunological and clinical point of view. Besides the level of pO2/FiO2, the immunological parameters that were the main responsible for the clusterization in responders were plasma levels of IL-2, IL-13, IL-1α, the absolute number of B cells as well as the memory unswitched B subset and among CD4^+^ T cells those TM expressing CD38 and PD1. Plasma levels of CXCL10, CXC3CL1, BAFF and the absolute number of different subpopulations of CD8^+^ T cells such as those EM expressing CD38, CD57 and HLA-DR were the major contributors for the clusterization of non-responders along PC1 (**Figure 5B**). Moreover, as previously reported, age, levels of D-dimer, SOFA score and levels of creatine are the major demographic/clinical features that are mostly associated to worse disease outcome and lack of response to therapy (2–6).

## Discussion

**Figure 6:**
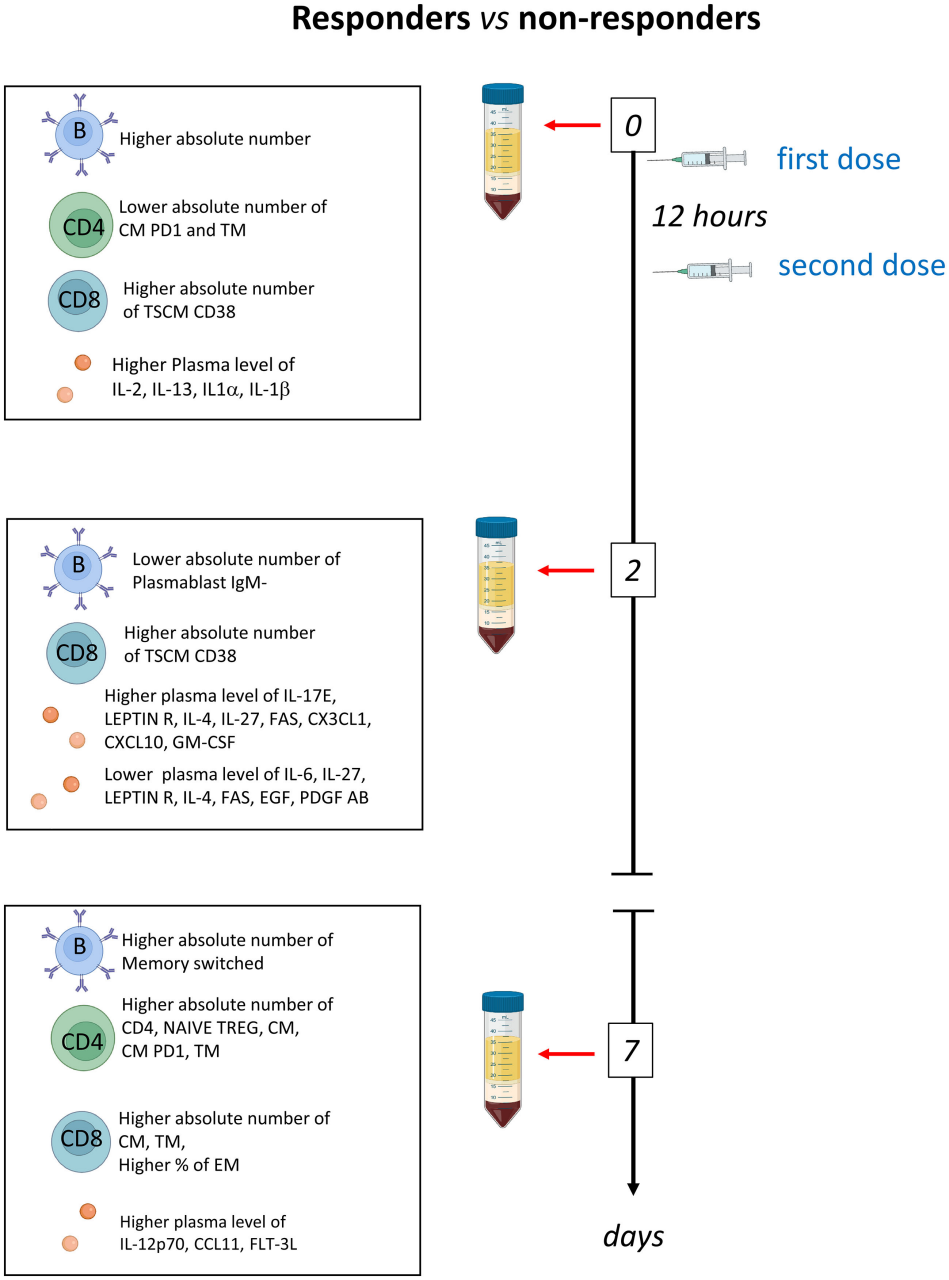
Schematic summary of the main results obtained before therapy (T0) and after two (T2) and 7 days (T7) in responder and non-responders. Tocilizumab was administered immediately after the first blood collection and 12 hours later.

Several observational and randomized studies, including TESEO, RECOVERY and REMAP-CAP, demonstrated that tocilizumab is effective in COVID-19 patients with hypoxemia and in need of oxygen therapy and that it improves survival in critically ill patients in intensive care units (ICUs) (15–17, 23). Despite transforming the treatment options for COVID-19, IL-6R inhibition is still ineffective in a fraction of patients. Thus, to identify possible differences between patients responding or not, we deeply investigated the humoral and adaptive immune compartments of COVID-19 patients undergoing tocilizumab treatment. We found that patients who respond to therapy, are characterized by high basal level of plasmatic IL-1β, IL-1α, IL-2, IL-13 and PDGF and lower level of CXCL10, higher number of B, CD4^+^ and CD8^+^ T cells which increase after therapy (**Figure 6**).

Tocilizumab is a monoclonal antibody that blocks interleukin-6 signaling, reducing downstream effects on inflammation and the immune response, which is largely used for years by rheumatologists. Tocilizumab binds to both soluble (s)IL-6R and membrane (m)IL-6R, thus inhibiting IL-6 binding to its receptors and leading to the blockade of the IL-6 signaling without interfering with other cytokines of the IL-6 family (24). IL-6 is essential for the maturation of B cells and it acts as a T cell survival factor driving T cell expansion under inflammatory conditions, but not during normal homeostasis (25, 26). IL-6 also inhibits TGF-β-induced Treg differentiation (27), and this reinforces its inflammatory aspect.

We found that, at the time of treatment initiation, COVID-19 patients who responded to tocilizumab had higher levels of several pro-inflammatory cytokines, including IL-1β, IL-1α and IFN-β. This is in agreement with a previous observation showing that the efficacy of tocilizumab is higher in COVID-19 patients with a hyperinflammatory state (28). Similarly, in patients with high IL-6, early administration of tocilizumab was associated with improvement in oxygenation, indicated as the ratio between arterial oxygen tension and fraction of inspired oxygen (29). In our cohort, IL-6 plasma levels were not different between patients responding or not to therapy at the time of treatment initiation, but in non-responders such levels increased after two days of therapy. In other conditions, including rheumatoid arthritis (RA) and Castleman disease, both serum IL-6 and sIL-6R increase in patients after administration of tocilizumab while the disease symptoms ameliorates (30). In this setting, it is likely that free serum IL-6 increases because IL-6R-mediated consumption of IL-6 is inhibited by the unavailability of tocilizumab-free IL-6R, and thus that serum IL-6 during the inhibition of its receptor by tocilizumab represents the actual endogenous production of IL-6 (30). In our cohort, IL-6 levels increased after two days of therapy in non-responders. We could hypothesize that, also in this case, plasma IL-6 levels represent the actual endogenous production of IL-6 and the true disease activity of patients when the causal factors of IL-6 overproduction are not adequately counterbalanced. Conversely, in responders, causal factors are neutralized and IL-6 level decreases by natural protein degradation.

Tocilizumab acts by inhibiting IL-6 and its autocrine activity on B cell growth, and is expected to inhibit the differentiation of B cells and the survival of plasma cells (31, 32). We found that tocilizumab affects B cell homeostasis in all COVID-19 patients, responding or not to therapy. The number of B cells, and in particular the number of naïve B cells and of memory switched B cells, increases after tocilizumab administration irrespective of response to treatment. In neuromyelitis optica spectrum disorder (NMOSD), tocilizumab treatment led to an increase in the number of naïve B cells and decreases in the number of memory B cells and antibody-secreting B cells (31). B cell phenotype and IgD^-^CD27^-^ memory B cells are also affected by tocilizumab in patients with RA (33). Concerning the response to tocilizumab, we found that responders have a higher basal number of B cells if compared to non-responders. In particular, patients with high number of memory switched and memory IgM only cells will more likely benefit from the treatment if compared to patients with lower numbers. In other settings, i.e. patients with RA who had an inadequate response to disease-modifying antirheumatic drugs and a TNF inhibitor, tocilizumab proved more effective than rituximab in B cell-poor but not in B cell-rich patients (34).

IL-6 also acts on T cells, and its inhibition has several implications both on CD4^+^ or CD8^+^ T cells. Responder patients have higher number of CD4^+^ and CD8^+^ T cells, with a particular increase of naïve and recently activated memory T cell population after therapy. In inflammation due to RA, tocilizumab contributes to induce the increase of protective Treg and to inhibit the Th17 phenotype (35–37). Six hallmarks describe the main alterations occurring in the early infection phase with SARS-CoV-2 and in the course of the disease, which predispose to severe illness. These six hallmarks, that we have observed in our previous studies, are: i) dysregulated type I IFN activity; ii) hyperinflammation; iii) lymphopenia; iv) lymphocyte impairment; v) dysregulated myeloid response; and vi) heterogeneous adaptive immunity to the virus (38). Concerning tocilizumab treatment, we found that patients responding to therapy were characterized by higher level of inflammation and higher levels of B cells and T cells if compared to those not responding, whereas non-responders were characterized by leukopenia. Moreover, patients who respond to therapy were characterized by a higher activation of the immune system (identified as the expression of recent markers of activation such as CD38 and PD1) and the increase of naïve T cells, together with the decrease of plasmablasts. Accumulating evidence is implicating immunosuppression in the development of severe COVID-19. Therefore, COVID-19 management should aim to reverse immunosuppression and prevent resultant opportunistic infections. In line with these observations, timing of tocilizumab administration plays a crucial role, as if patient is experiencing immunoparalysis, or, on the other hand, has poor or negligible symptoms, tocilizumab is likely unable to induce a clinically useful response.

We acknowledge that our study has some limitations. The observational nature and the limited number of patients represent a first weakness of this study. This is a single centre study, and the results may not be generalizable to the wider population. However, the longitudinal design with a close follow-up represents a remarkable strength together with the accurate assessment of cellular immune response and the comprehensive analysis of soluble factors, at different time points, before treatment and after two and seven days of treatment. To our knowledge this is the first study to date in which the immunological differences between COVID-19 patients responding or not to tocilizumab have been evaluated.

In conclusion, we found that tocilizumab has pleiotropic effects and that clinical response to this drug remain heterogenous. However, our data suggest that it is possible to identify patients who will respond to treatment and that the administration of tocilizumab is able to restore the immune balance through the re-establishment of different cell populations affected by SARS-COV-2 infection, highlighting the importance of temporal examination of the pathological features from the diagnosis.

## Methods

### Patients and study design

This is a case-control, longitudinal, single-center study, approved by the local Ethical Committee (Comitato Etico dell’Area Vasta Emilia Nord, protocol number 177/2020, March 11th, 2020) and by the University Hospital Committee (Direzione Sanitaria dell’Azienda Ospedaliero-Universitaria di Modena, protocol number 7531, March 11th, 2020). Each participant provided informed consent according to Helsinki Declaration, and all uses of human material have been approved by the same Committees. A total of 23 patients with severe COVID-19 was included in the study; they were all treated with tocilizumab. Thirteen patients were responders to therapy, whereas ten patients were non-responders. All patients had severe pneumonia, defined as at least one of the following: (i) presence of a respiratory rate of 30 or more breaths per minute, (ii) peripheral blood oxygen saturation (SaO_2_) of less than 93% in room air, (iii) a ratio of arterial oxygen partial pressure (PaO2) to fractional inspired oxygen (FiO_2_) of less than 300 mm Hg in room air, and (iv) lung infiltrates of more than 50% within 24–48 h, according to Chinese management guidelines for COVID-19 (version 6.0) (39). All patients received standard of care treatment at the time of hospital admission according to the regional COVID-19 guidelines of Emilia Romagna. In addition to the standard of care treatment, patients also received tocilizumab treatment. Depending on the availability of specific formulation at time of treatment, twelve patients received tocilizumab by intravenous administration at 8 mg/kg bodyweight (up to a maximum of 800 mg) given twice, 12 hours apart, while eleven patients received it by subcutaneous route at a dose of 162 mg administered in two simultaneous doses, one in each thigh (i.e., 324 mg in total). This particular subcutaneous approach was used to mimic, as much as possible, the pharmacokinetic activity of the intravenous formulation in order to achieve similar levels of drug exposure. After the administration of tocilizumab, patients were divided into 2 groups based on its clinical effects: 13 did not required invasive mechanical ventilation and were classified as responder, meanwhile 10 required invasive mechanical ventilation and were classified as non-responder. Three patients of the non-responder group died between 13 and 29 days after admission. We recorded demographic data, medical history, and main laboratory findings from each patient. For details, see **Supplementary Table 1**. These data are referred to hospital admission, before starting therapy (T0).

### Blood collection and isolation of mononuclear cells

Up to 20 mL of blood were collected from each patient in vacuettes containing ethylenediamine-tetraacetic acid (EDTA). Blood was immediately processed. Isolation of peripheral blood mononuclear cells (PBMC) was performed using ficoll-hypaque according to standard procedures. PBMC were stored in liquid nitrogen in fetal bovine serum (FBS) supplemented with 10% dimethyl sulfoxide (DMSO). Plasma was stored at -80°C until use. Measurements were taken from individual patients; in the case of plasma, each measurement was performed in duplicate and only the mean was considered and shown.

### Quantification of cytokine plasma levels

The plasma levels of 62 molecular species were quantified using a Luminex platform (Human Cytokine Discovery, R&D System, Minneapolis, MN) for the simultaneous detection of the following molecules: G-CSF, PDGF-AA, EGF, PDGF-AB/BB, VEGF, GM-CSF, FGF, GRZB, IL-1A, IL-1RA, IL-2, IL-27, IL-4, IL-6, IL-10, IL-13, TNF, IL-17C, IL-11, IL-18, IL-23, IL-6RA, IL-19, IFN-α, IFN-β, IL-3, IL-5, IL-7, IL-12p70, IL-15, IL-33, TGF- β, IFN- γ, IL-1B, IL-17, IL-17E, CCL3, CCL11, CCL20, CXCCL1, CXCL2, CCL5, CCL2, CCL4, CCL19, CXCL1, CXCL10, PD-L1, FLT-3, TACI, FAS, LEPTIN R, APRIL, OPN, BAFF, LEPTIN, BMP4, CD40 LIGAND, FAS LIGAND, BMP7, BMP2, TRAIL, according to the manufacturer’s instruction. Data represent the mean of two technical replicates.

### Polychromatic flow cytometry

All data obtained by flow cytometric assays have been produced according to the state-of-the-art technologies, as described (40).

### B cell characterization

Thawed PBMC were washed twice with RPMI 1640 supplemented with 10% fetal bovine serum and 1% each of l-glutamine, sodium pyruvate, nonessential amino acids, antibiotics, 0.1 M HEPES, 55 μM β-mercaptoethanol and 0.02 mg/ml DNAse. Thawed PBMC were stained with viability marker Promokine IR-840 (PromoCell GmbH, Heidelberg, Germany) for 20 min at room temperature in PBS. One million PBMC were washed with FACS buffer and stained with DuraClone IM B cells containing the following lyophilized directly conjugated mAbs: IgD-FITC, CD21-PE, CD19-ECD, CD27-PC7, CD24-APC, CD38-AF750, IgM-PB, CD45-KrO. Cells were washed with FACS buffer and acquired at Cytoflex LX flow cytometer (Beckman Coulter, Hialeah, FL). A minimum of 500,000 cells was acquired on a CytoFLEX LX flow cytometer (Beckman Coulter).

### T cell characterization

Thawed PBMC were washed twice with RPMI 1640 supplemented with 10% fetal bovine serum and 1% each of l-glutamine, sodium pyruvate, nonessential amino acids, anti- biotics, 0.1 M HEPES, 55 μM β-mercaptoethanol and 0.02 mg/ml DNAse. Up to 1 million PBMC were stained with the Duraclone IM T cell panel (Beckman Coulter) added with another five fluorescent mAbs and a marker of cell viability. Along with side and forward scatter signals, signals were obtained from different fluorochrome-labeled mAbs, i.e., CD45 conjugated with Krome Orange, CD3 APC-A750, CD4-APC, CD8-AF700, CD27-PC7, CD57-Pacific Blue, CD279(PD1)-PC5.5, CD28-ECD, CCR7-PE, CD45RA-FITC, HLA-DR-BUV661, CD127-BV650, CD25-BV785, CD95-BUV395, CD38-BUV496, and PromoFluor-840 (Promokine, PromoCell, Heidelberg, Germany). A minimum of 500,000 cells per sample were acquired on a CytoFLEX LX flow cytometer (Beckman Coulter).

### Representation of high parameter flow cytometry

Flow Cytometry Standard (FCS) 3.0 files were imported into FlowJo software version X (Becton Dickinson, San Joseè, CA), and analyzed by standard gating to eliminate aggregates and dead cells, and to identify CD3^+^CD4^+^ T cells, CD3^+^CD8^+^ T cells (**Supplementary Figures 7**) and CD19^+^ B cells (**Supplementary Figures 8**). Data were exported for further analysis in R, by following a script that makes use of Bioconductor libraries and R statistical packages (CATALYST 1.10.1). The script is available at: https://github.com/HelenaLC/CATALYST). The selection of cofactor for data transformation was checked on Cytobank premium version (see: cytobank.org). Metaclustering (K=25) was performed by using FlowSOM algorithm. Dimensional reduction was performed using UMAP algorithm.

B cell UMAP graphs stratified by patient at time T0, T2 and T7 are reported in **Supplementary Figures 9A–C**, respectively. B cell projection of UMAP graphs stratified by patient showing the FlowSOM clusters at time T0, T2, T7 are reported in **Supplementary Figures 10A–C**, respectively. CD4^+^ T cell UMAP graphs stratified by patient at time T0, T2 and T7 are reported in **Supplementary Figures 11A–C**, respectively. CD4^+^ T cell projection of UMAP graphs stratified by patient showing the FlowSOM clusters at time T0, T2, T7 are reported in **Supplementary Figures 12A–C**, respectively. CD8^+^ T cell UMAP graphs stratified by patient at time T0, T2 and T7 are reported in **Supplementary Figures 13A–C**, respectively. CD8^+^ T cell projection of UMAP graphs stratified by patient showing the FlowSOM clusters at time T0, T2, T7 are reported in **Supplementary Figures 14A–C**, respectively.

### Principal component analysis

We have then investigated the role of B and T cells along with several clinical and biochemical parameters. For this purpose, we used the principal component analysis (PCA), a dimension reduction method that retains the characteristics of a data set that contribute most to its variance, by keeping lower order principal components (PCs) and ignoring the others (41). PCA uses an orthogonal transformation to collapse the dataset containing correlated parameters to a smaller set of linearly uncorrelated variables known as PCs, such that each PC is a weighted combination of all the markers. We performed this analysis to test whether subject classification was possible based on the B and T cell profile, cytokines and/or clinical data. Thus, PCA was carried out on the clinical events (responder or non-responder), and a dataset comprising 137 parameters that included age, blood pH, pCO_2_ (mmHg), pO_2_ (mmHg), sO_2_ (%), pO_2_/FiO_2_ ratio, ALT (U/L), total bilirubin (mg/dL), CK (U/L), creatinine (mg/dL), D-Dimer (ng/mL), Hb (g/dL), white blood cells, WBC (N/μL), red blood cells, RBC (10^6^/μL), INR (ratio), LDH (U/L), CRP (mg/dL), platelets (10^9^/L), respiratory rate (breaths/minute), systolic blood pressure (mmHg), heart rate (beats/minute), SOFA score, different absolute numbers of B-cell populations (naïve, memory switched, memory IgM-only, transitional, memory unswitched, plasmablasts, exhausted, surface-) and T-cell subpopulations.

### Statistical analysis

High-dimensional cytometric analysis was performed by using differential discovery in high-dimensional cytometry *via* high-resolution clustering. Quantitative variables were compared using Two-way ANOVA with Bonferroni correction. Data are represented as individual values, means, and standard errors of the mean. Statistical analyses were performed using Prism 8.4.3 (GraphPad Software Inc., La Jolla, USA).

## Data availability statement

The raw data supporting the conclusions of this article will be made available by the authors, without undue reservation.

## Ethics statement

The studies involving human participants were reviewed and approved by Comitato Etico dell’Area Vasta Emilia Nord, protocol number 177/2020 Direzione Sanitaria dell’Azienda Ospedaliero-Universitaria di Modena, protocol number 7531. The patients/participants provided their written informed consent to participate in this study.

## Author contributions

LG, SDB, MMa and DT carried out experiments and drafted the figures. AP, RB, AN, LF, MMa, DT collected and stored PBMC samples. LG, SDB drafted and revised the figures and the tables. MMe, GG, CM, SB, MG, FC, AM followed patients. SDB, LG, DT, MMa performed bioinformatic and statistical analyses. LG, SDB, CM and AC conceived the study and wrote the manuscript. All authors contributed to the article and approved the submitted version.

## References

[1] Verity R Okell LC Dorigatti I Winskill P Whittaker C Imai N. Estimates of the severity of coronavirus disease 2019: a model-based analysis. Lancet Infect Dis (2020) 20(6):669–77. doi: 10.1016/S1473-3099(20)30243-7 PMC7158570 32240634

[2] De Biasi S Lo Tartaro D Meschiari M Gibellini L Bellinazzi C Borella R. Expansion of plasmablasts and loss of memory b cells in peripheral blood from COVID-19 patients with pneumonia. Eur J Immunol (2020) 50(9):1283–94. doi: 10.1002/eji.202048838 32910469

[3] De Biasi S Meschiari M Gibellini L Bellinazzi C Borella R Fidanza L. Marked T cell activation, senescence, exhaustion and skewing towards TH17 in patients with COVID-19 pneumonia. Nat Commun (2020) 11(1):3434. doi: 10.1038/s41467-020-17292-4 32632085 PMC7338513

[4] Gibellini L De Biasi S Paolini A Borella R Boraldi F Mattioli M. Altered bioenergetics and mitochondrial dysfunction of monocytes in patients with COVID-19 pneumonia. EMBO Mol Med (2020) 12(12):e13001. doi: 10.15252/emmm.202013001 33078545 PMC7645870

[5] Gibellini L De Biasi S Meschiari M Gozzi L Paolini A Borella R. Plasma cytokine atlas reveals the importance of TH2 polarization and interferons in predicting COVID-19 severity and survival. Front Immunol (2022) 13:842150. doi: 10.3389/fimmu.2022.842150 35386702 PMC8979161

[6] Lo Tartaro D Neroni A Paolini A Borella R Mattioli M Fidanza L. Molecular and cellular immune features of aged patients with severe COVID-19 pneumonia. Commun Biol (2022) 5(1):590. doi: 10.1038/s42003-022-03537-z 35710943 PMC9203559

[7] Kishimoto T. Interleukin-6: from basic science to medicine–40 years in immunology. Annu Rev Immunol (2005) 23:1–21. doi: 10.1146/annurev.immunol.23.021704.115806 15771564

[8] Murakami M Kamimura D Hirano T. Pleiotropy and specificity: insights from the interleukin-6 family of cytokines. Immunity (2019) 50(4):812–31. doi: 10.1016/j.immuni.2019.03.027 30995501

[9] Wolf J Rose-John S Garbers C. Interleukin-6 and its receptors: a highly regulated and dynamic system. Cytokine (2014) 70(1):11–20. doi: 10.1016/j.cyto.2014.05.024 24986424

[10] Choy EH De Benedetti F Takeuchi T Hashizume M John MR Kishimoto T. Translating IL-6 biology into effective treatments. Nat Rev Rheumatol (2020) 16(6):335–45. doi: 10.1038/s41584-020-0419-z PMC7178926 32327746

[11] Rose-John S Winthrop K Calabrese L. The role of IL-6 in host defence against infections: immunobiology and clinical implications. Nat Rev Rheumatol (2017) 13(7):399–409. doi: 10.1038/nrrheum.2017.83 28615731

[12] Chen LYC Hoiland RL Stukas S Wellington CL Sekhon MS. Confronting the controversy: interleukin-6 and the COVID-19 cytokine storm syndrome. Eur Respir J (2020) 56(4):2003006. doi: 10.1183/13993003.03006-2020 32883678 PMC7474149

[13] Chen LYC Hoiland RL Stukas S Wellington CL Sekhon MS. Assessing the importance of interleukin-6 in COVID-19. Lancet Respir Med (2021) 9(2):e13. doi: 10.1016/S2213-2600(20)30600-7 33460569 PMC7836242

[14] Guo C Li B Ma H Wang X Cai P Yu Q. Single-cell analysis of two severe COVID-19 patients reveals a monocyte-associated and tocilizumab-responding cytokine storm. Nat Commun (2020) 11(1):3924. doi: 10.1038/s41467-020-17834-w 32764665 PMC7413381

[15] Group RC. Tocilizumab in patients admitted to hospital with COVID-19 (RECOVERY): a randomised, controlled, open-label, platform trial. Lancet (2021) 397(10285):1637–45. doi: 10.1016/S0140-6736(21)00676-0 PMC8084355 33933206

[16] Guaraldi G Meschiari M Cozzi-Lepri A Milic J Tonelli R Menozzi M. Tocilizumab in patients with severe COVID-19: a retrospective cohort study. Lancet Rheumatol (2020) 2(8):e474–84. doi: 10.1016/S2665-9913(20)30173-9 PMC7314456 32835257

[17] Investigators R-C Gordon AC Mouncey PR Al-Beidh F Rowan KM Nichol AD. Interleukin-6 receptor antagonists in critically ill patients with covid-19. N Engl J Med (2021) 384(16):1491–502. doi: 10.1056/NEJMoa2100433 PMC7953461 33631065

[18] Borcoman E Kanjanapan Y Champiat S Kato S Servois V Kurzrock R. Novel patterns of response under immunotherapy. Ann Oncol (2019) 30(3):385–96. doi: 10.1093/annonc/mdz003 30657859

[19] De Biasi S Gibellini L Lo Tartaro D Puccio S Rabacchi C Mazza EMC. Circulating mucosal-associated invariant T cells identify patients responding to anti-PD-1 therapy. Nat Commun (2021) 12(1):1669. doi: 10.1038/s41467-021-21928-4 33723257 PMC7961017

[20] Scalzo-Inguanti K Plebanski M. CD38 identifies a hypo-proliferative IL-13-secreting CD4+ T-cell subset that does not fit into existing naive and memory phenotype paradigms. Eur J Immunol (2011) 41(5):1298–308. doi: 10.1002/eji.201040726 21469087

[21] Jubel JM Barbati ZR Burger C Wirtz DC Schildberg FA. The role of PD-1 in acute and chronic infection. Front Immunol (2020) 11:487. doi: 10.3389/fimmu.2020.00487 32265932 PMC7105608

[22] Ahn E Araki K Hashimoto M Li W Riley JL Cheung J. Role of PD-1 during effector CD8 T cell differentiation. Proc Natl Acad Sci U.S.A. (2018) 115(18):4749–54. doi: 10.1073/pnas.1718217115 PMC5939075 29654146

[23] Abidi E El Nekidy WS Alefishat E Rahman N Petroianu GA El-Lababidi R. Tocilizumab and COVID-19: timing of administration and efficacy. Front Pharmacol (2022) 13:825749. doi: 10.3389/fphar.2022.825749 35250575 PMC8894855

[24] Mihara M Kasutani K Okazaki M Nakamura A Kawai S Sugimoto M. Tocilizumab inhibits signal transduction mediated by both mIL-6R and sIL-6R, but not by the receptors of other members of IL-6 cytokine family. Int Immunopharmacol (2005) 5(12):1731–40. doi: 10.1016/j.intimp.2005.05.010 16102523

[25] Li B Jones LL Geiger TL. IL-6 promotes T cell proliferation and expansion under inflammatory conditions in association with low-level RORgammat expression. J Immunol (2018) 201(10):2934–46. doi: 10.4049/jimmunol.1800016 PMC6324200 30315140

[26] Roldan E Brieva JA. Terminal differentiation of human bone marrow cells capable of spontaneous and high-rate immunoglobulin secretion: role of bone marrow stromal cells and interleukin 6. Eur J Immunol (1991) 21(11):2671–7. doi: 10.1002/eji.1830211105 1936115

[27] Bettelli E Carrier Y Gao W Korn T Strom TB Oukka M. Reciprocal developmental pathways for the generation of pathogenic effector TH17 and regulatory T cells. Nature (2006) 441(7090):235–8. doi: 10.1038/nature04753 16648838

[28] Rodriguez-Bano J Pachon J Carratala J Ryan P Jarrin I Yllescas M. Treatment with tocilizumab or corticosteroids for COVID-19 patients with hyperinflammatory state: a multicentre cohort study (SAM-COVID-19). Clin Microbiol Infect (2021) 27(2):244–52. doi: 10.1016/j.cmi.2020.08.010 PMC7449935 32860964

[29] Galvan-Roman JM Rodriguez-Garcia SC Roy-Vallejo E Marcos-Jimenez A Sanchez-Alonso S Fernandez-Diaz C. IL-6 serum levels predict severity and response to tocilizumab in COVID-19: an observational study. J Allergy Clin Immunol (2021) 147(1):72–80 e78. doi: 10.1016/j.jaci.2020.09.018 33010257 PMC7525244

[30] Nishimoto N Terao K Mima T Nakahara H Takagi N Kakehi T. Mechanisms and pathologic significances in increase in serum interleukin-6 (IL-6) and soluble IL-6 receptor after administration of an anti-IL-6 receptor antibody, tocilizumab, in patients with rheumatoid arthritis and castleman disease. Blood (2008) 112(10):3959–64. doi: 10.1182/blood-2008-05-155846 18784373

[31] Liu Y Zhang H Zhang TX Yuan M Du C Zeng P. Effects of tocilizumab therapy on circulating b cells and T helper cells in patients with neuromyelitis optica spectrum disorder. Front Immunol (2021) 12:703931. doi: 10.3389/fimmu.2021.703931 34394101 PMC8360623

[32] Snir A Kessel A Haj T Rosner I Slobodin G Toubi E. Anti-IL-6 receptor antibody (tocilizumab): a b cell targeting therapy. Clin Exp Rheumatol (2011) 29(4):697–700. doi: 10.1136/ard.2010.149005.1 21813064

[33] Moura RA Quaresma C Vieira AR Goncalves MJ Polido-Pereira J Romao VC. B-cell phenotype and IgD-CD27- memory b cells are affected by TNF-inhibitors and tocilizumab treatment in rheumatoid arthritis. PloS One (2017) 12(9):e0182927. doi: 10.1371/journal.pone.0182927 28886017 PMC5590747

[34] Humby F Durez P Buch MH Lewis MJ Rizvi H Rivellese F. Rituximab versus tocilizumab in anti-TNF inadequate responder patients with rheumatoid arthritis (R4RA): 16-week outcomes of a stratified, biopsy-driven, multicentre, open-label, phase 4 randomised controlled trial. Lancet (2021) 397(10271):305–17. doi: 10.1016/S0140-6736(20)32341-2 PMC7829614 33485455

[35] Kikuchi J Hashizume M Kaneko Y Yoshimoto K Nishina N Takeuchi T. Peripheral blood CD4(+)CD25(+)CD127(low) regulatory T cells are significantly increased by tocilizumab treatment in patients with rheumatoid arthritis: increase in regulatory T cells correlates with clinical response. Arthritis Res Ther (2015) 17:10. doi: 10.1186/s13075-015-0526-4 25604867 PMC4332922

[36] Pesce B Soto L Sabugo F Wurmann P Cuchacovich M Lopez MN. Effect of interleukin-6 receptor blockade on the balance between regulatory T cells and T helper type 17 cells in rheumatoid arthritis patients. Clin Exp Immunol (2013) 171(3):237–42. doi: 10.1111/cei.12017 PMC3569529 23379428

[37] Samson M Audia S Janikashvili N Ciudad M Trad M Fraszczak J. Brief report: inhibition of interleukin-6 function corrects Th17/Treg cell imbalance in patients with rheumatoid arthritis. Arthritis Rheum (2012) 64(8):2499–503. doi: 10.1002/art.34477 22488116

[38] Mazzoni A Salvati L Maggi L Annunziato F Cosmi L. Hallmarks of immune response in COVID-19: exploring dysregulation and exhaustion. Semin Immunol (2021) 55:101508. doi: 10.1016/j.smim.2021.101508 34728121 PMC8547971

[39] Zhou F Yu T Du R Fan G Liu Y Liu Z. Clinical course and risk factors for mortality of adult inpatients with COVID-19 in wuhan, China: a retrospective cohort study. Lancet (2020) 395(10229):1054–62. doi: 10.1016/S0140-6736(20)30566-3 PMC7270627 32171076

[40] Cossarizza A Chang HD Radbruch A Abrignani S Addo R Akdis M. Guidelines for the use of flow cytometry and cell sorting in immunological studies (third edition). Eur J Immunol (2021) 51:2708–3145. doi: 10.1002/eji.202170126 34910301 PMC11115438

[41] Borella R De Biasi S Paolini A Boraldi F Lo Tartaro D Mattioli M. Metabolic reprograming shapes neutrophil functions in severe COVID-19. Eur J Immunol (2022) 52(3):484–502. doi: 10.1002/eji.202149481 34870329

