# Supplementary material for: Prognostic immune markers identifying patients with severe COVID-19 who respond to tocilizumab
Source: Front Immunol. 2023 May 5;14:1123807. doi: 10.3389/fimmu.2023.1123807 (PMC10196248; doi:10.3389/fimmu.2023.1123807)
Supplement: Supplementary file 1 [file Presentation_1.pptx]

## Slide 1
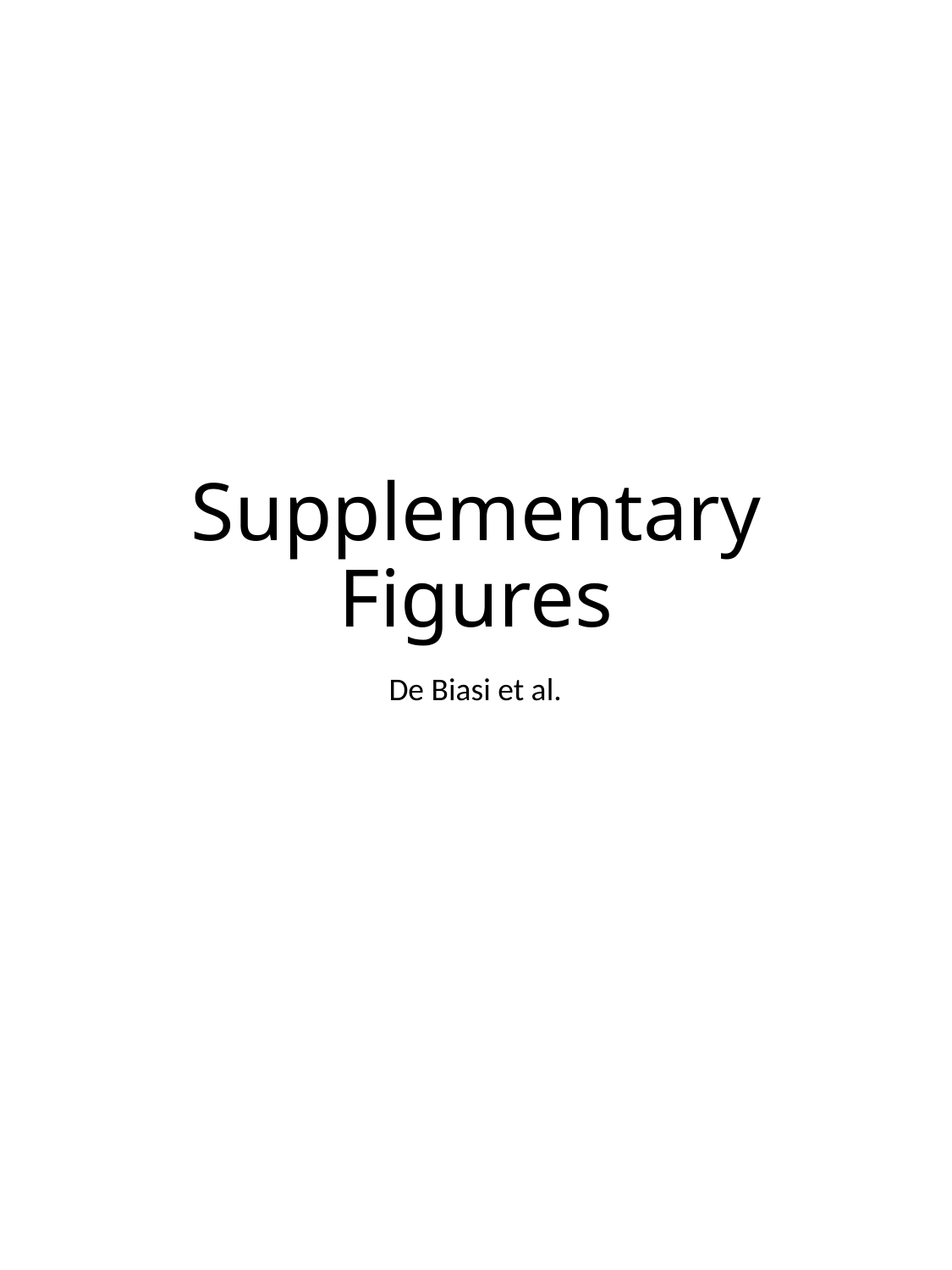

# Supplementary Figures
De Biasi et al.

## Slide 2
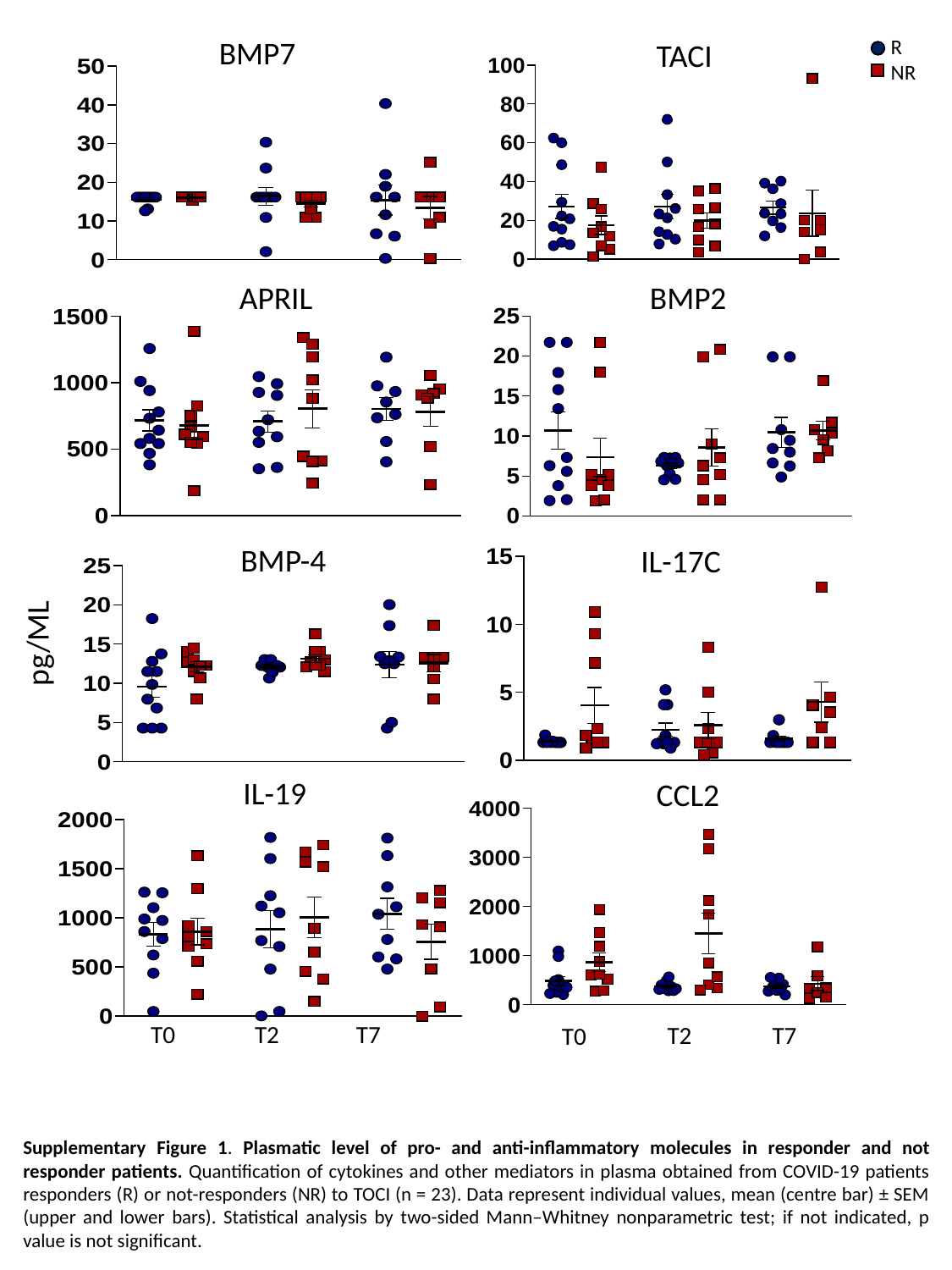

BMP7
TACI
APRIL
BMP2
BMP-4
IL-17C
IL-19
CCL2
R
NR
pg/ML
T7
T2
T0
T7
T2
T0
Supplementary Figure 1. Plasmatic level of pro- and anti-inflammatory molecules in responder and not responder patients. Quantification of cytokines and other mediators in plasma obtained from COVID-19 patients responders (R) or not-responders (NR) to TOCI (n = 23). Data represent individual values, mean (centre bar) ± SEM (upper and lower bars). Statistical analysis by two-sided Mann–Whitney nonparametric test; if not indicated, p value is not significant.

## Slide 3
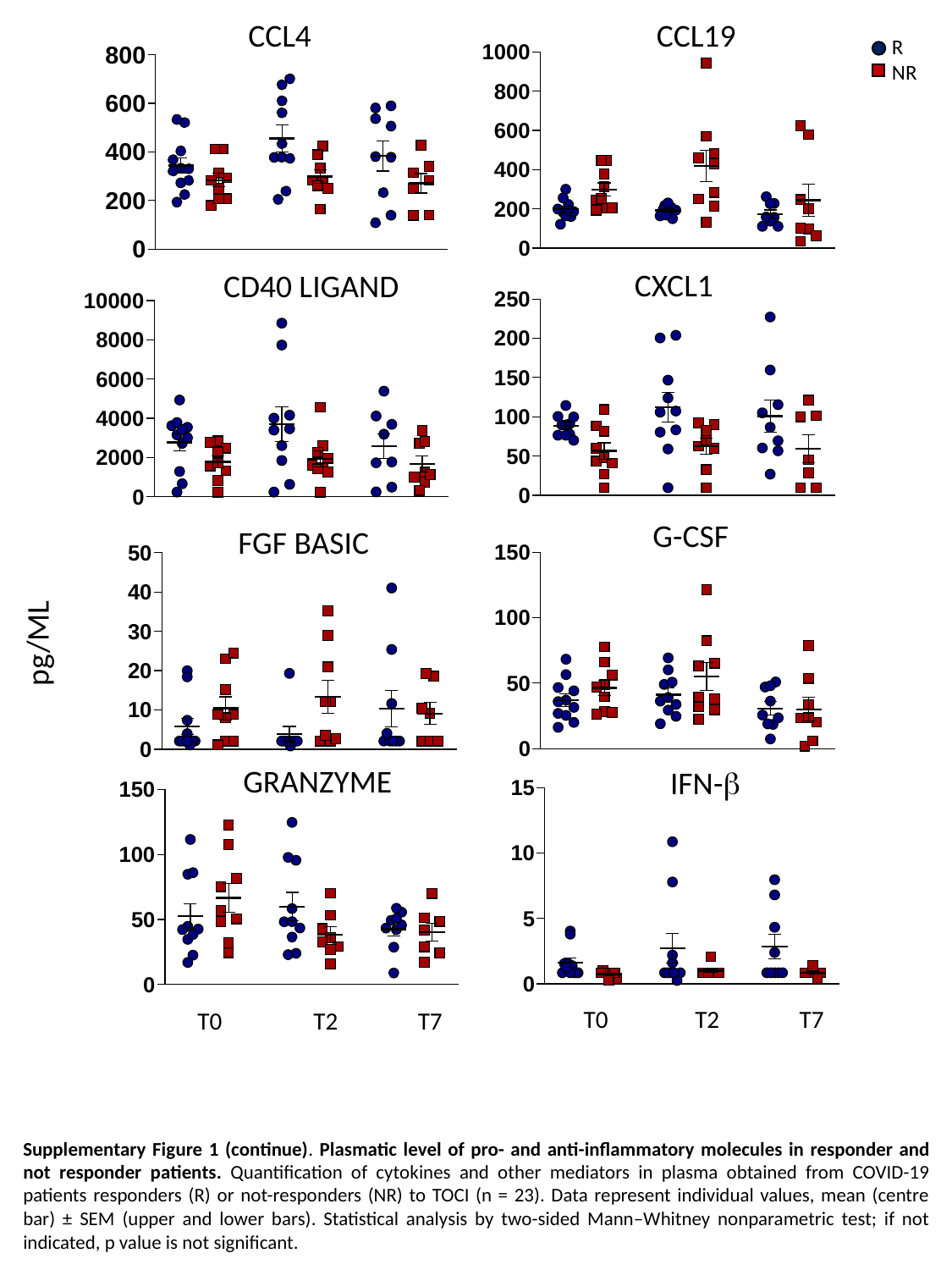

CCL19
CCL4
CXCL1
CD40 LIGAND
G-CSF
FGF BASIC
GRANZYME
IFN-b
R
NR
pg/ML
T7
T2
T0
T7
T2
T0
Supplementary Figure 1 (continue). Plasmatic level of pro- and anti-inflammatory molecules in responder and not responder patients. Quantification of cytokines and other mediators in plasma obtained from COVID-19 patients responders (R) or not-responders (NR) to TOCI (n = 23). Data represent individual values, mean (centre bar) ± SEM (upper and lower bars). Statistical analysis by two-sided Mann–Whitney nonparametric test; if not indicated, p value is not significant.

## Slide 4
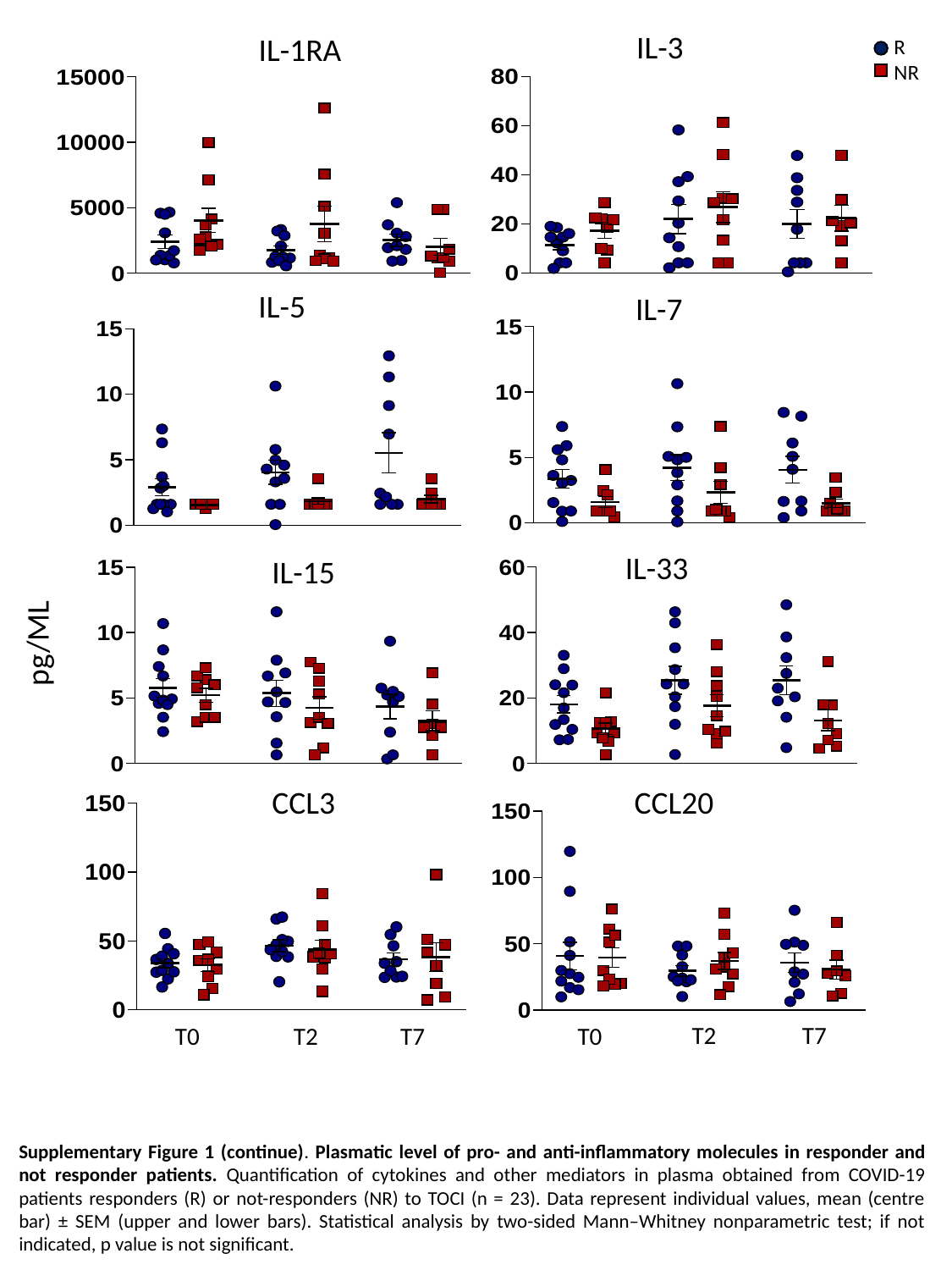

IL-3
IL-1RA
IL-5
IL-7
IL-33
IL-15
CCL3
CCL20
R
NR
pg/ML
T7
T2
T0
T7
T2
T0
Supplementary Figure 1 (continue). Plasmatic level of pro- and anti-inflammatory molecules in responder and not responder patients. Quantification of cytokines and other mediators in plasma obtained from COVID-19 patients responders (R) or not-responders (NR) to TOCI (n = 23). Data represent individual values, mean (centre bar) ± SEM (upper and lower bars). Statistical analysis by two-sided Mann–Whitney nonparametric test; if not indicated, p value is not significant.

## Slide 5
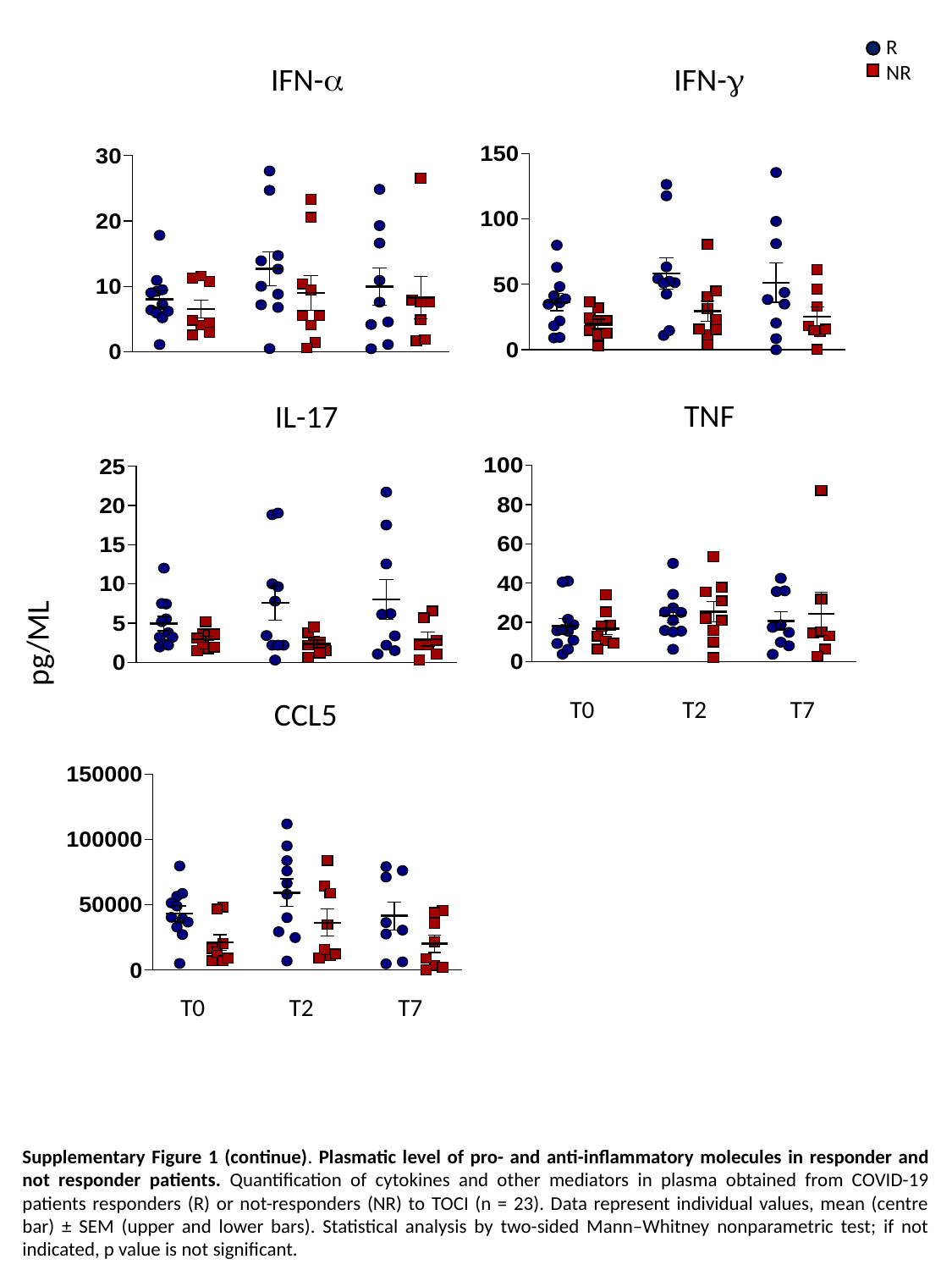

R
NR
IFN-a
IFN-g
TNF
IL-17
CCL5
pg/ML
T7
T2
T0
T7
T2
T0
Supplementary Figure 1 (continue). Plasmatic level of pro- and anti-inflammatory molecules in responder and not responder patients. Quantification of cytokines and other mediators in plasma obtained from COVID-19 patients responders (R) or not-responders (NR) to TOCI (n = 23). Data represent individual values, mean (centre bar) ± SEM (upper and lower bars). Statistical analysis by two-sided Mann–Whitney nonparametric test; if not indicated, p value is not significant.

## Slide 6
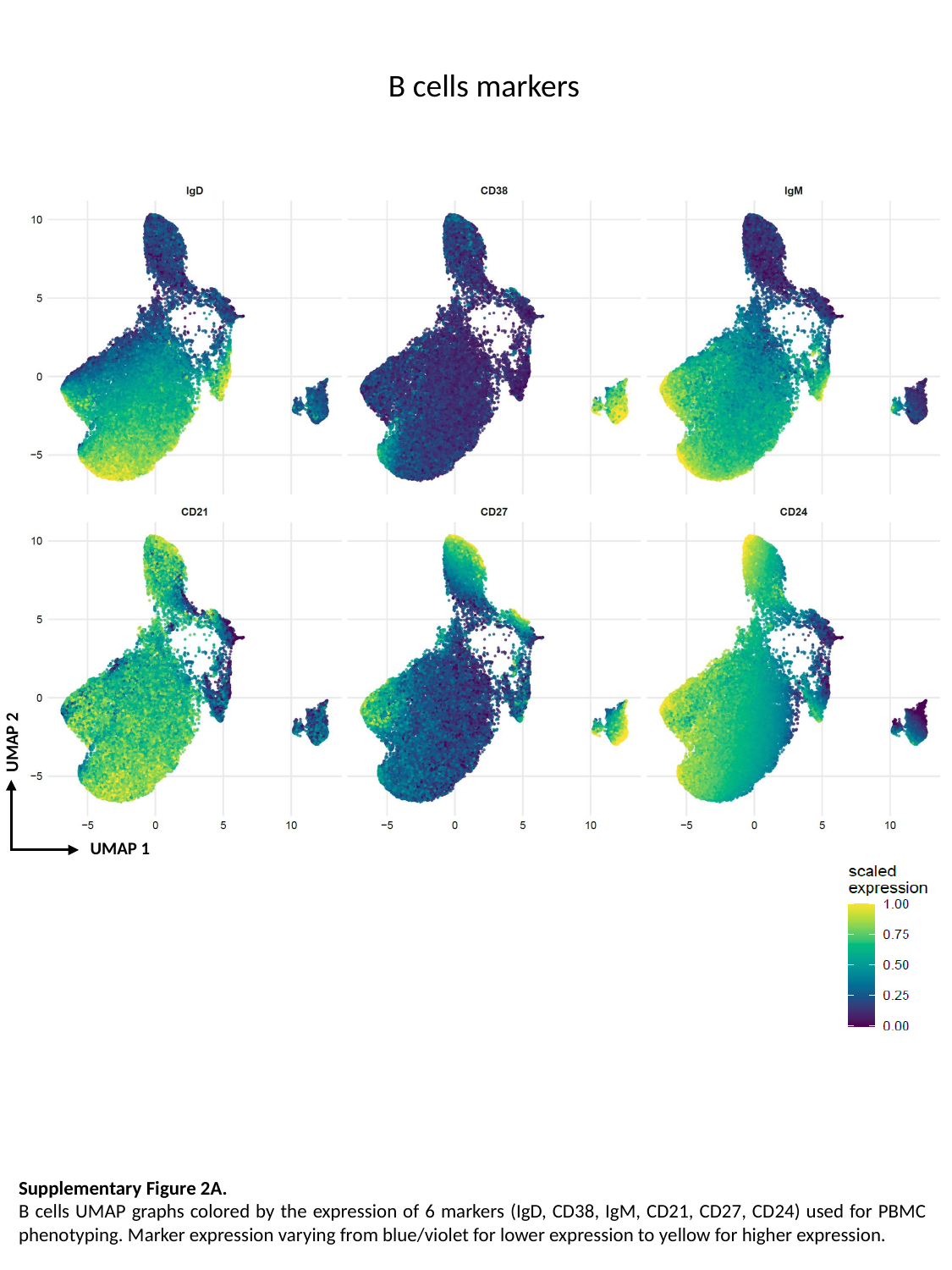

B cells markers
UMAP 2
UMAP 1
Supplementary Figure 2A.
B cells UMAP graphs colored by the expression of 6 markers (IgD, CD38, IgM, CD21, CD27, CD24) used for PBMC phenotyping. Marker expression varying from blue/violet for lower expression to yellow for higher expression.

## Slide 7
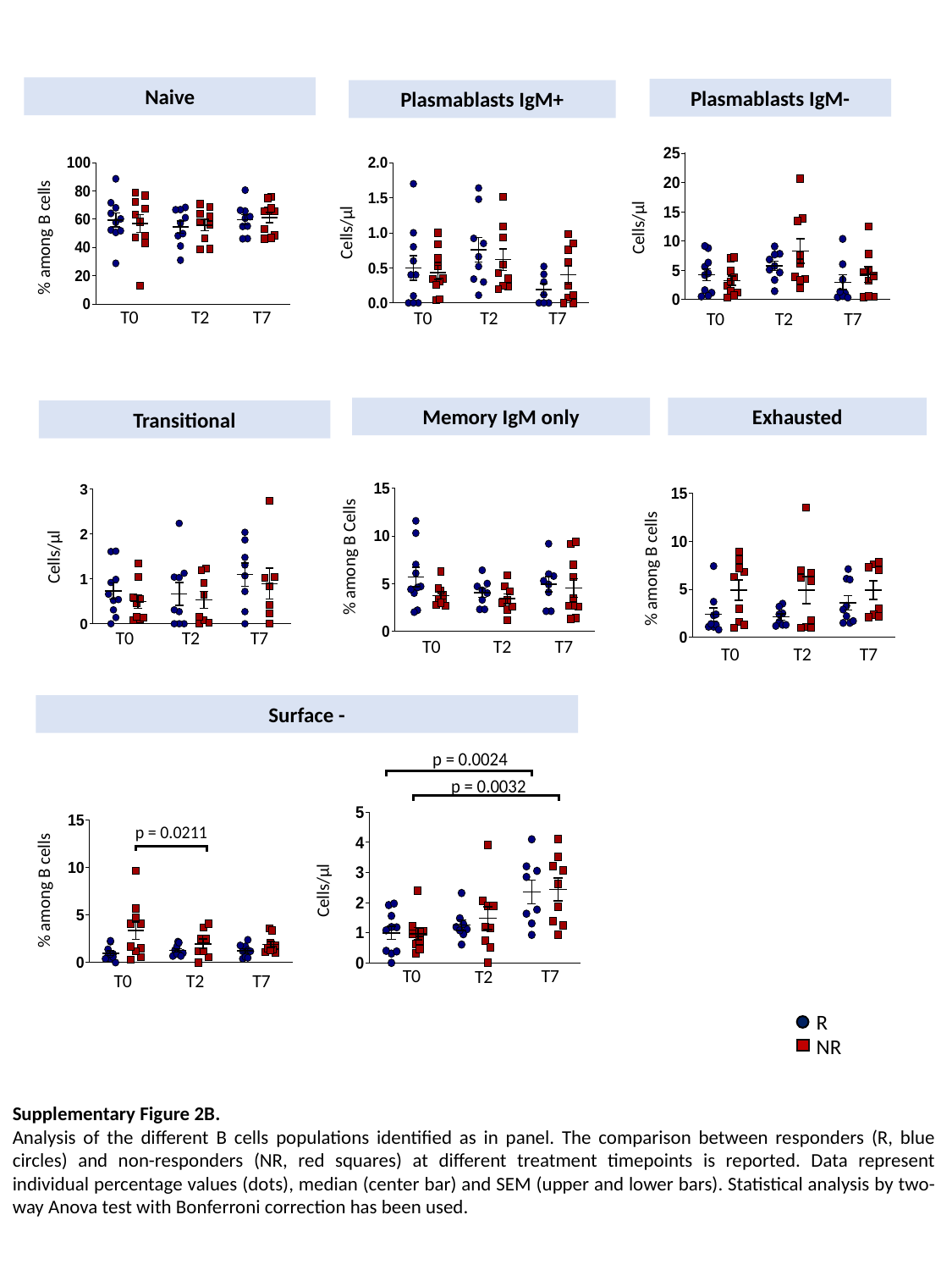

Naive
Plasmablasts IgM-
Plasmablasts IgM+
Cells/µl
Cells/µl
% among B cells
T0
T7
T2
T0
T7
T2
T0
T7
T2
Memory IgM only
Exhausted
Transitional
Cells/µl
% among B Cells
% among B cells
T0
T7
T2
T0
T7
T2
T0
T7
T2
Surface -
Cells/µl
% among B cells
T0
T7
T2
T0
T7
T2
R
NR
Supplementary Figure 2B.
Analysis of the different B cells populations identified as in panel. The comparison between responders (R, blue circles) and non-responders (NR, red squares) at different treatment timepoints is reported. Data represent individual percentage values (dots), median (center bar) and SEM (upper and lower bars). Statistical analysis by two-way Anova test with Bonferroni correction has been used.

## Slide 8
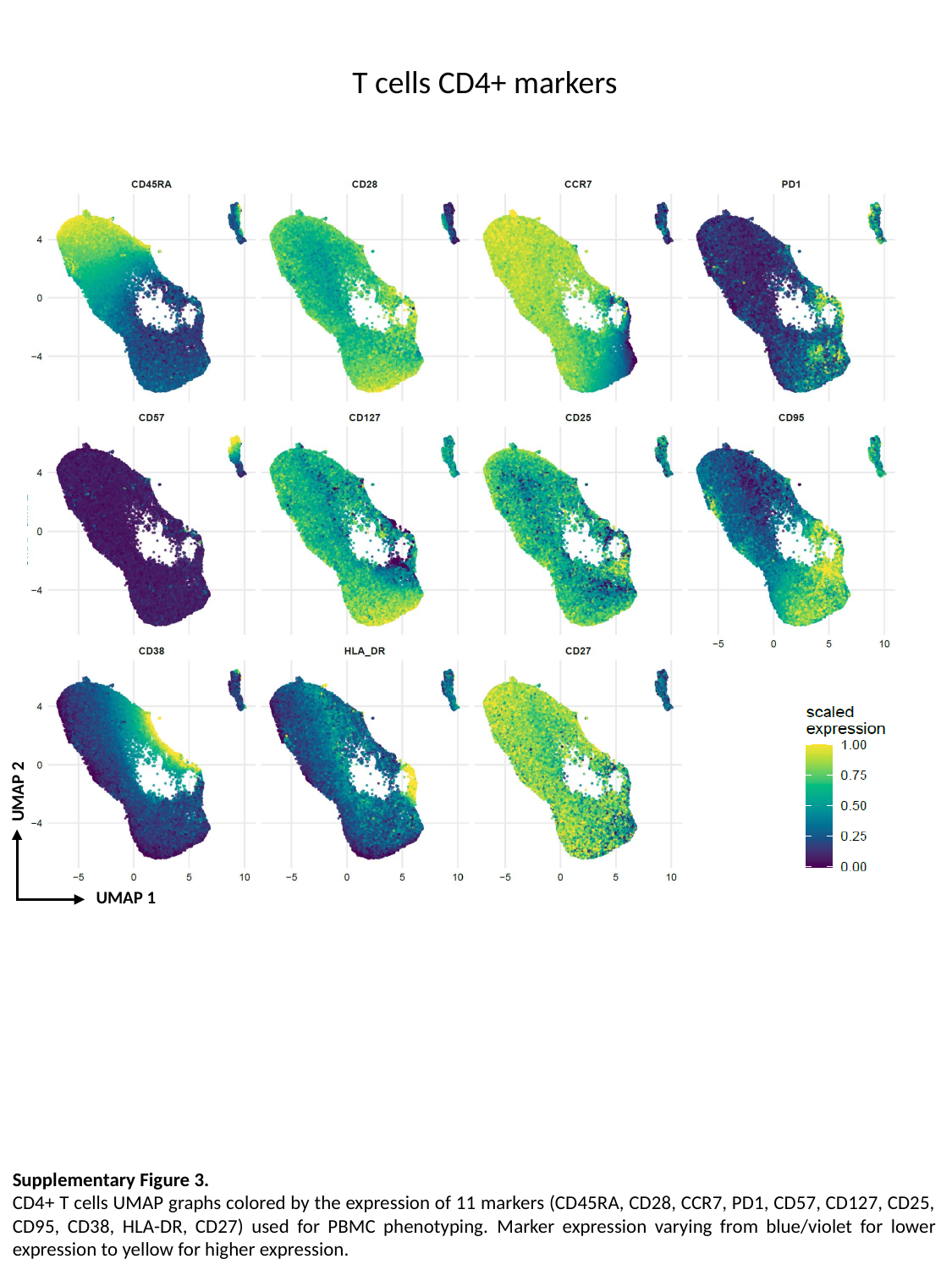

T cells CD4+ markers
UMAP 2
UMAP 1
Supplementary Figure 3.
CD4+ T cells UMAP graphs colored by the expression of 11 markers (CD45RA, CD28, CCR7, PD1, CD57, CD127, CD25, CD95, CD38, HLA-DR, CD27) used for PBMC phenotyping. Marker expression varying from blue/violet for lower expression to yellow for higher expression.

## Slide 9
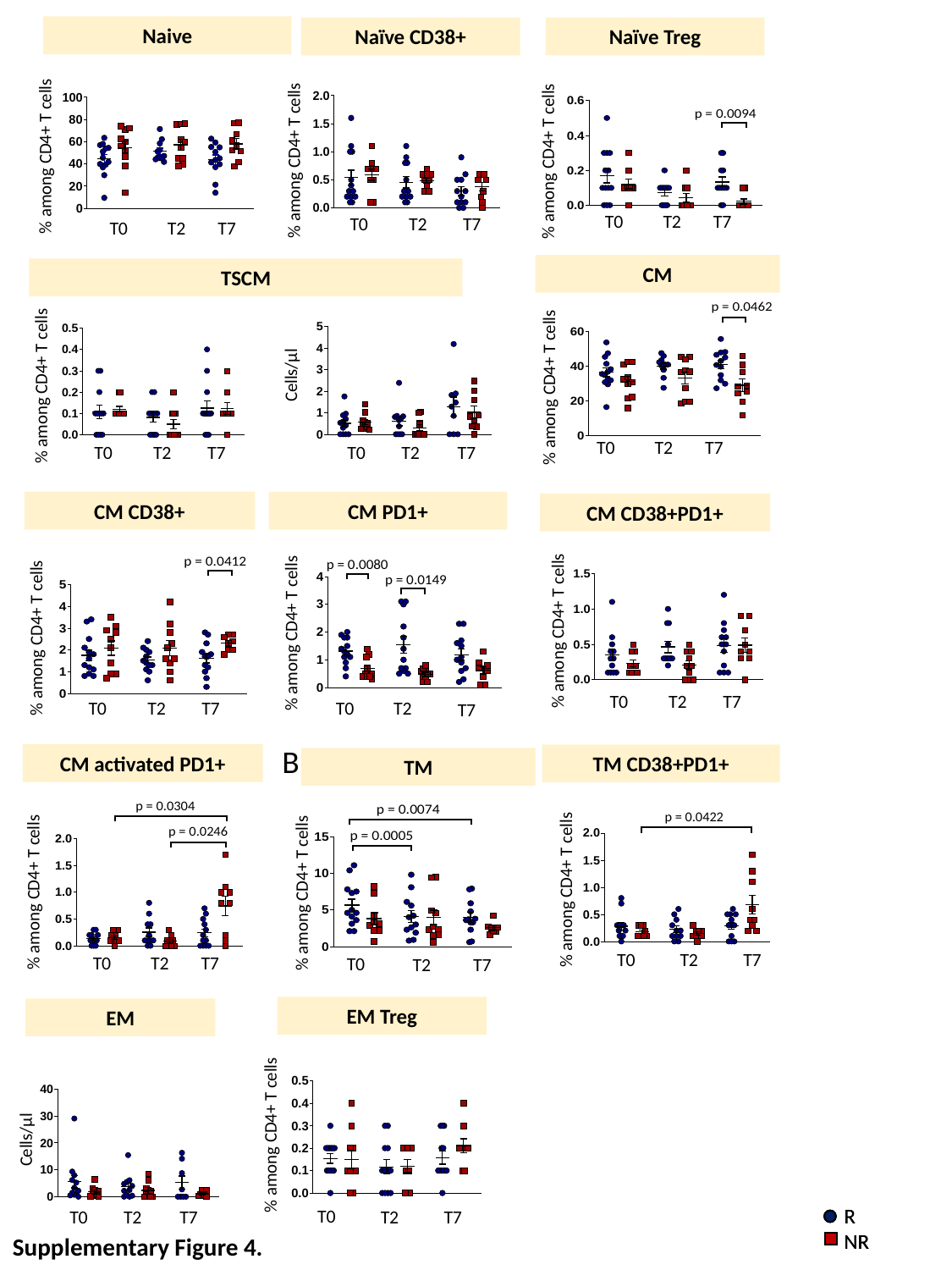

Naive
Naïve CD38+
Naïve Treg
% among CD4+ T cells
% among CD4+ T cells
% among CD4+ T cells
T0
T7
T2
T0
T7
T2
T0
T7
T2
CM
TSCM
Cells/µl
% among CD4+ T cells
% among CD4+ T cells
T0
T7
T2
T0
T7
T2
T0
T7
T2
CM CD38+
CM PD1+
CM CD38+PD1+
% among CD4+ T cells
% among CD4+ T cells
% among CD4+ T cells
T0
T7
T2
T0
T7
T2
T0
T2
T7
B
CM activated PD1+
TM CD38+PD1+
TM
% among CD4+ T cells
% among CD4+ T cells
% among CD4+ T cells
T0
T2
T7
T0
T7
T2
T0
T2
T7
EM Treg
EM
% among CD4+ T cells
Cells/µl
R
NR
R
NR
T0
T0
T7
T2
T7
T2
Supplementary Figure 4.

## Slide 10
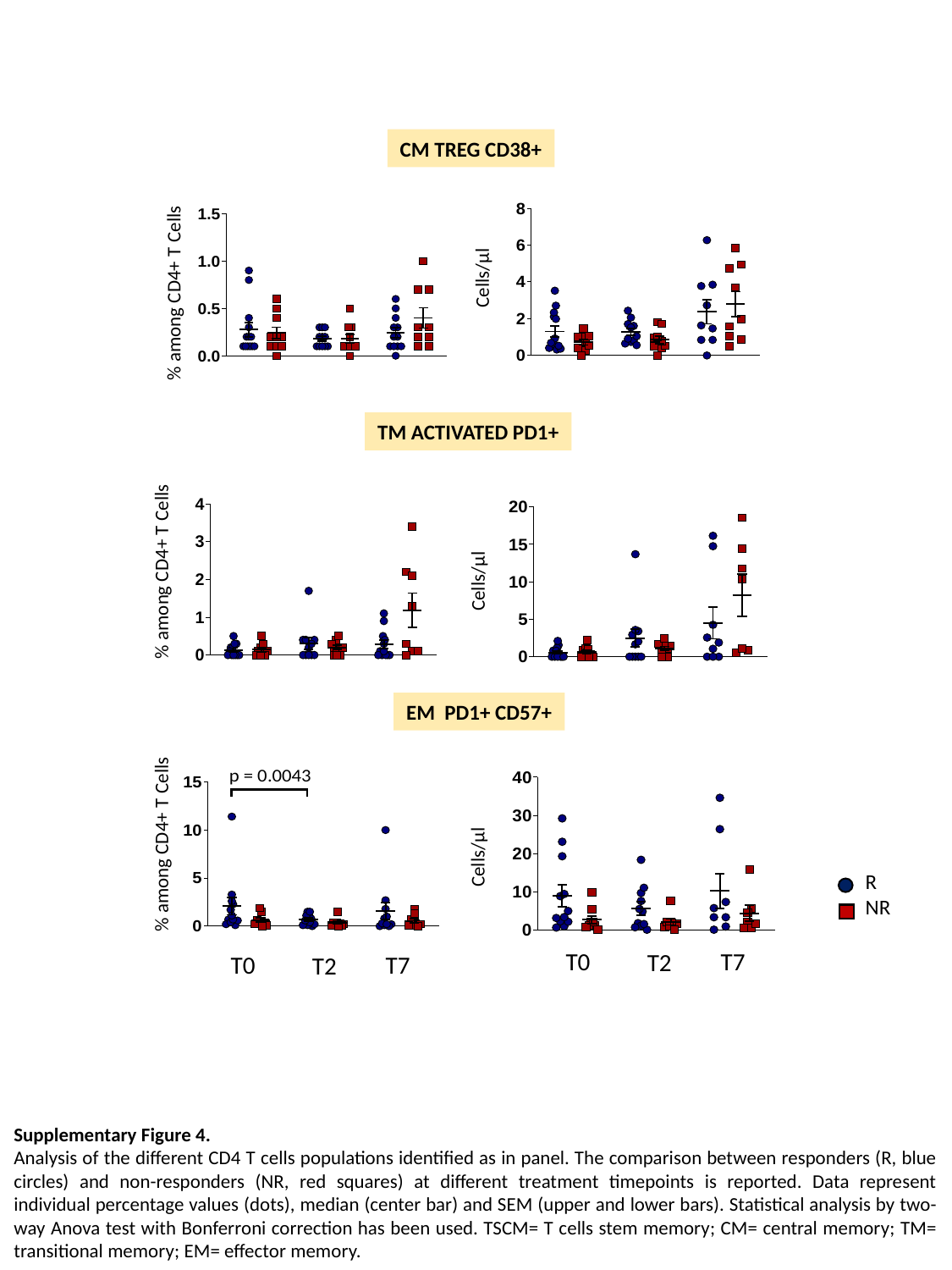

CM TREG CD38+
Cells/µl
% among CD4+ T Cells
TM ACTIVATED PD1+
% among CD4+ T Cells
Cells/µl
EM PD1+ CD57+
% among CD4+ T Cells
Cells/µl
R
NR
T0
T7
T2
T0
T7
T2
Supplementary Figure 4.
Analysis of the different CD4 T cells populations identified as in panel. The comparison between responders (R, blue circles) and non-responders (NR, red squares) at different treatment timepoints is reported. Data represent individual percentage values (dots), median (center bar) and SEM (upper and lower bars). Statistical analysis by two-way Anova test with Bonferroni correction has been used. TSCM= T cells stem memory; CM= central memory; TM= transitional memory; EM= effector memory.

## Slide 11
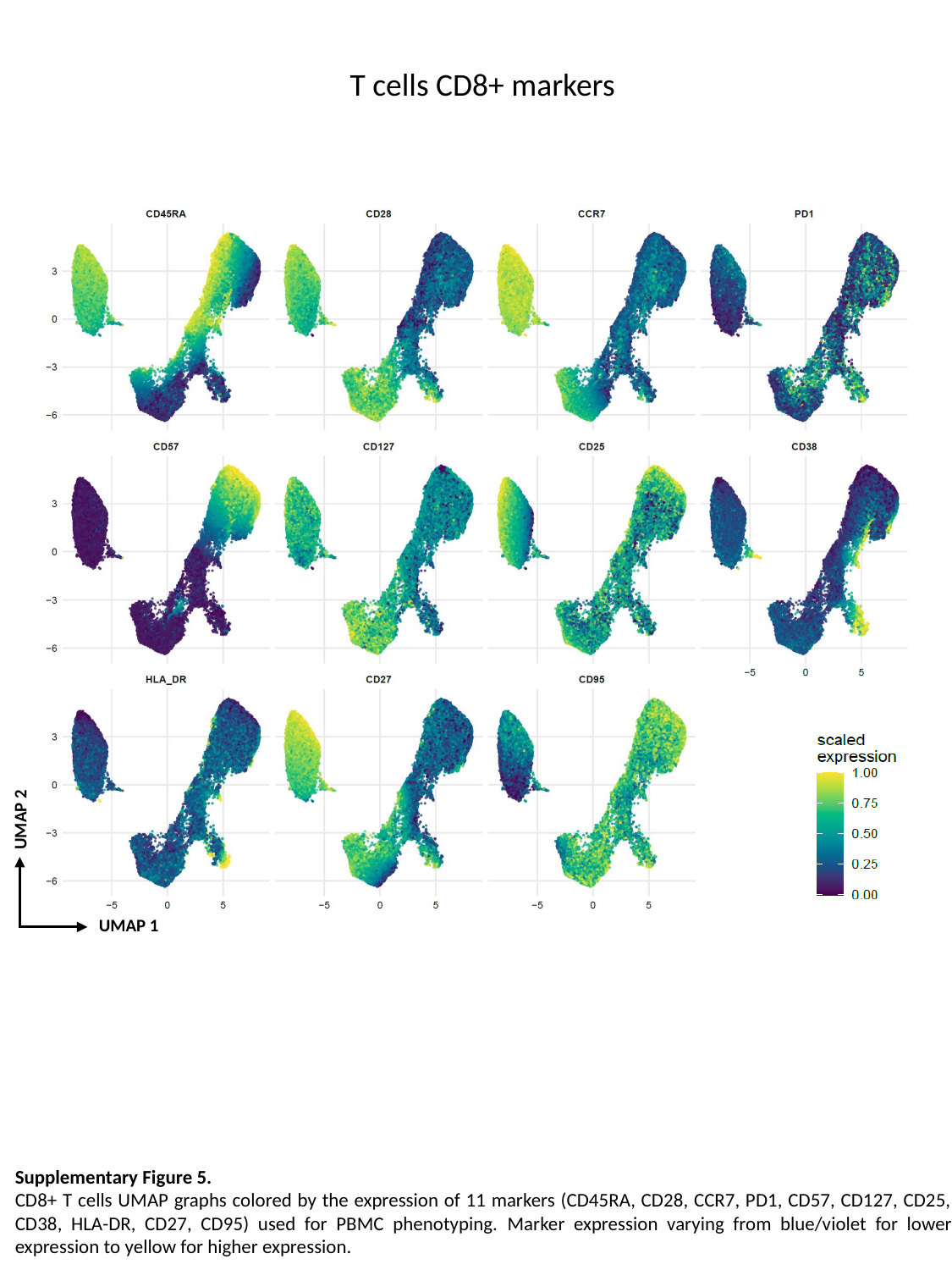

T cells CD8+ markers
UMAP 2
UMAP 1
Supplementary Figure 5.
CD8+ T cells UMAP graphs colored by the expression of 11 markers (CD45RA, CD28, CCR7, PD1, CD57, CD127, CD25, CD38, HLA-DR, CD27, CD95) used for PBMC phenotyping. Marker expression varying from blue/violet for lower expression to yellow for higher expression.

## Slide 12
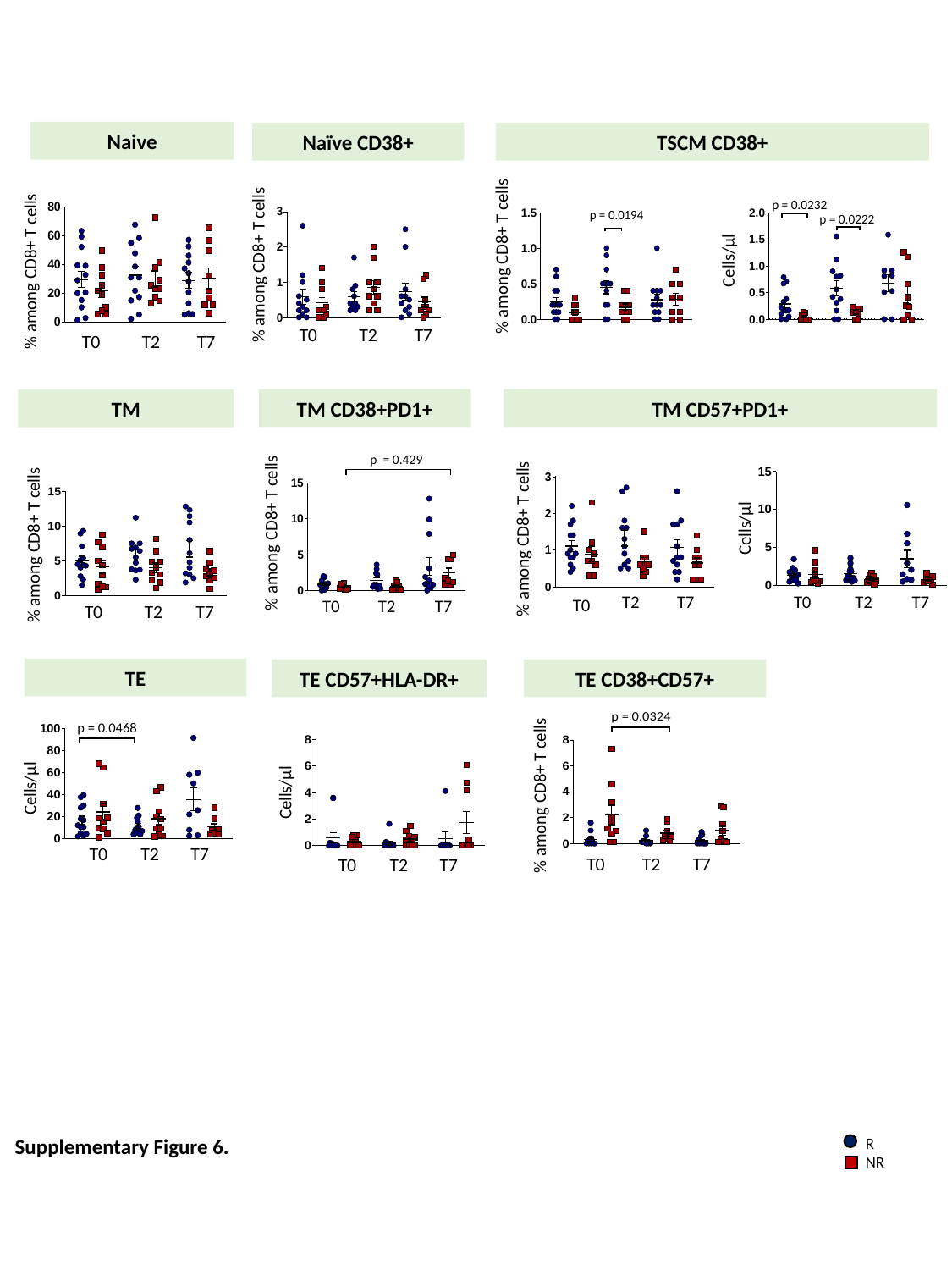

Naive
Naïve CD38+
TSCM CD38+
Cells/µl
% among CD8+ T cells
% among CD8+ T cells
% among CD8+ T cells
T0
T2
T7
T0
T2
T7
TM CD38+PD1+
TM CD57+PD1+
TM
Cells/µl
% among CD8+ T cells
% among CD8+ T cells
% among CD8+ T cells
T2
T7
T0
T2
T7
T0
T0
T2
T7
T0
T2
T7
TE
TE CD57+HLA-DR+
TE CD38+CD57+
Cells/µl
Cells/µl
% among CD8+ T cells
T0
T2
T7
T0
T2
T7
T0
T2
T7
Supplementary Figure 6.
R
NR

## Slide 13
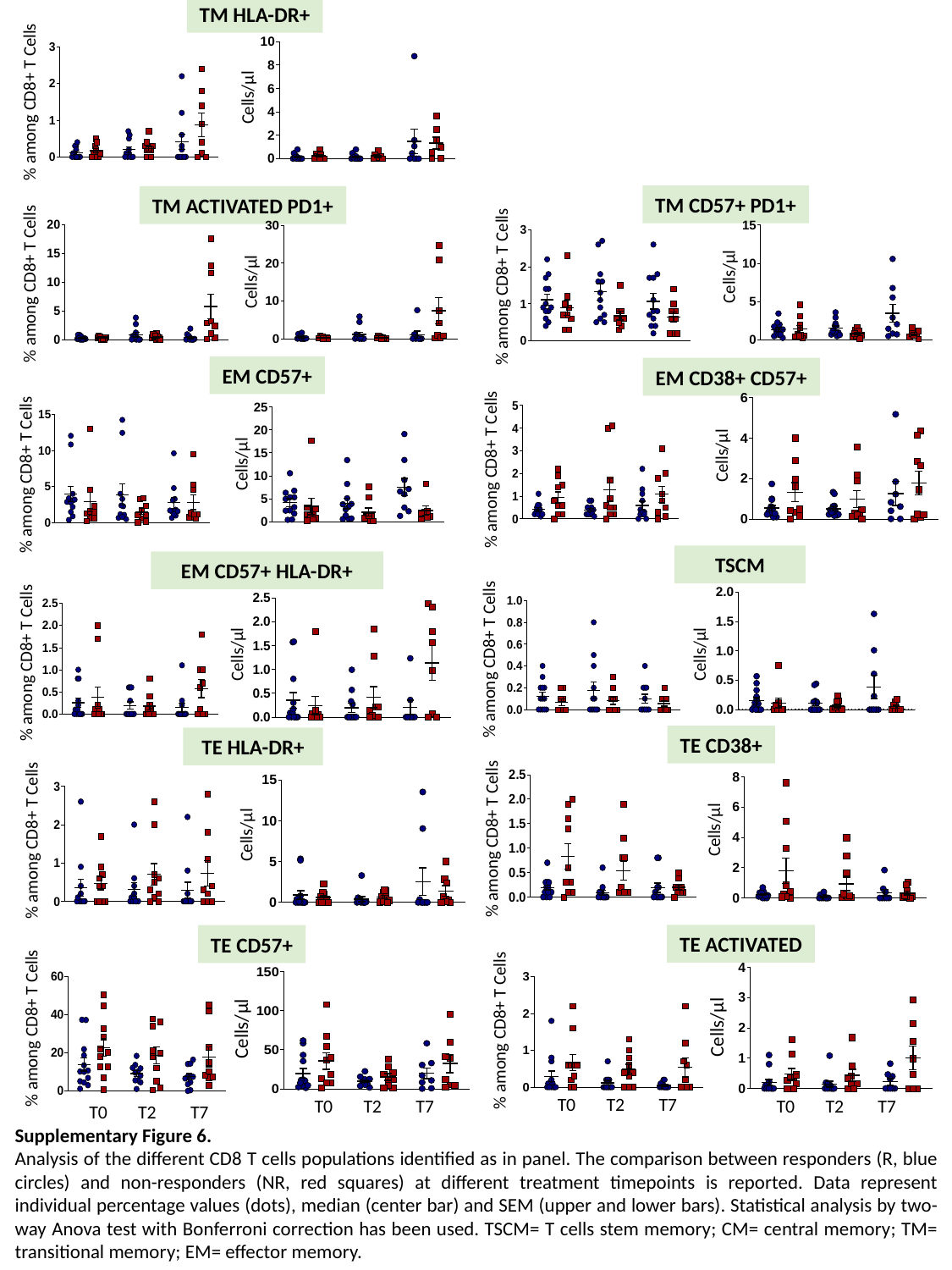

TM HLA-DR+
Cells/µl
% among CD8+ T Cells
TM CD57+ PD1+
TM ACTIVATED PD1+
Cells/µl
Cells/µl
% among CD8+ T Cells
% among CD8+ T Cells
EM CD57+
EM CD38+ CD57+
Cells/µl
Cells/µl
% among CD8+ T Cells
% among CD8+ T Cells
TSCM
EM CD57+ HLA-DR+
Cells/µl
Cells/µl
% among CD8+ T Cells
% among CD8+ T Cells
TE CD38+
TE HLA-DR+
Cells/µl
Cells/µl
% among CD8+ T Cells
% among CD8+ T Cells
TE ACTIVATED
TE CD57+
Cells/µl
Cells/µl
% among CD8+ T Cells
% among CD8+ T Cells
T0
T2
T7
T0
T2
T7
T0
T2
T7
T0
T2
T7
Supplementary Figure 6.
Analysis of the different CD8 T cells populations identified as in panel. The comparison between responders (R, blue circles) and non-responders (NR, red squares) at different treatment timepoints is reported. Data represent individual percentage values (dots), median (center bar) and SEM (upper and lower bars). Statistical analysis by two-way Anova test with Bonferroni correction has been used. TSCM= T cells stem memory; CM= central memory; TM= transitional memory; EM= effector memory.

## Slide 14
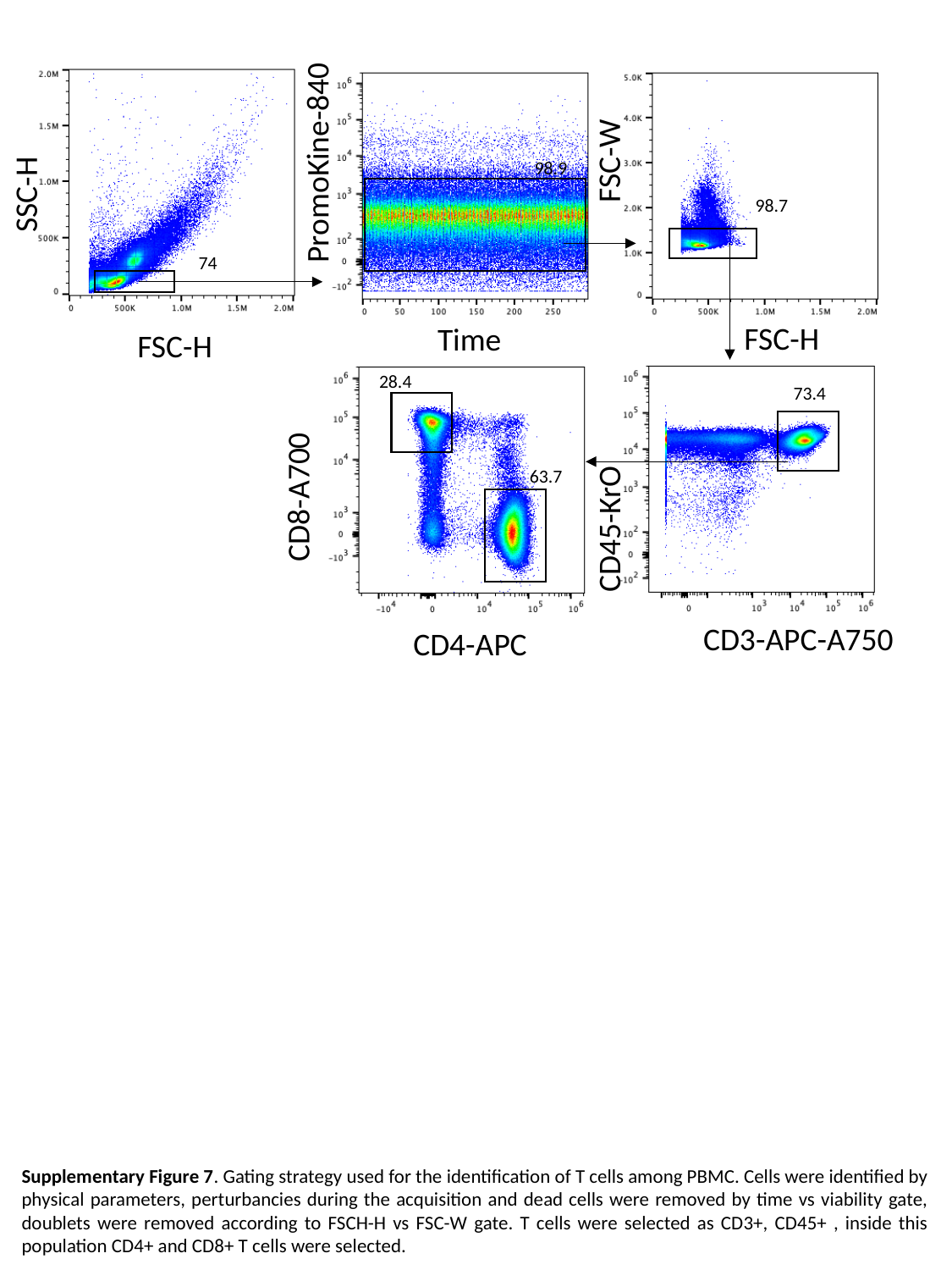

FSC-W
PromoKine-840
98.9
SSC-H
98.7
74
FSC-H
Time
FSC-H
28.4
73.4
63.7
CD8-A700
CD45-KrO
CD3-APC-A750
CD4-APC
Supplementary Figure 7. Gating strategy used for the identification of T cells among PBMC. Cells were identified by physical parameters, perturbancies during the acquisition and dead cells were removed by time vs viability gate, doublets were removed according to FSCH-H vs FSC-W gate. T cells were selected as CD3+, CD45+ , inside this population CD4+ and CD8+ T cells were selected.

## Slide 15
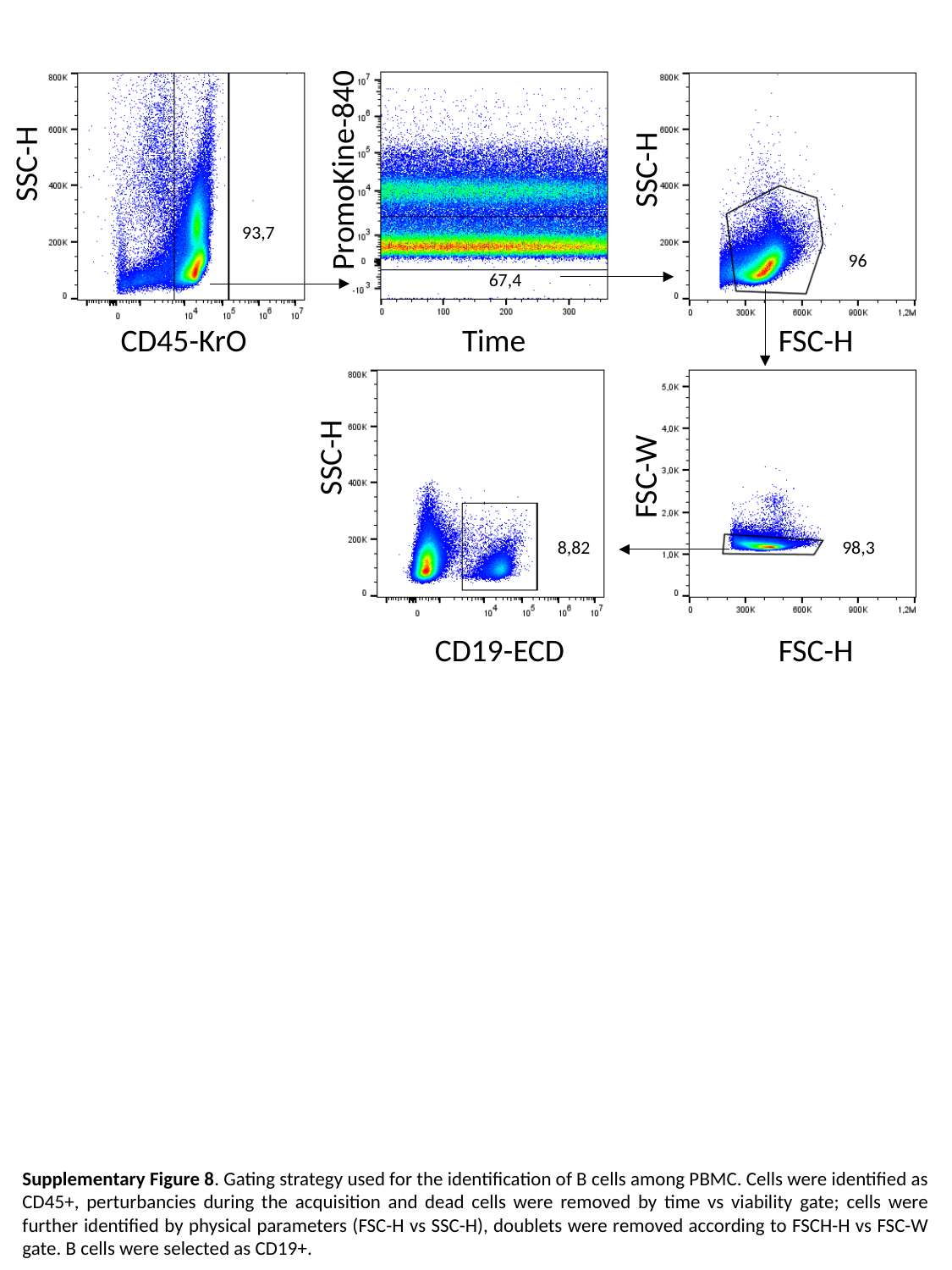

SSC-H
SSC-H
PromoKine-840
93,7
96
67,4
CD45-KrO
Time
FSC-H
SSC-H
FSC-W
8,82
98,3
CD19-ECD
FSC-H
Supplementary Figure 8. Gating strategy used for the identification of B cells among PBMC. Cells were identified as CD45+, perturbancies during the acquisition and dead cells were removed by time vs viability gate; cells were further identified by physical parameters (FSC-H vs SSC-H), doublets were removed according to FSCH-H vs FSC-W gate. B cells were selected as CD19+.

## Slide 16
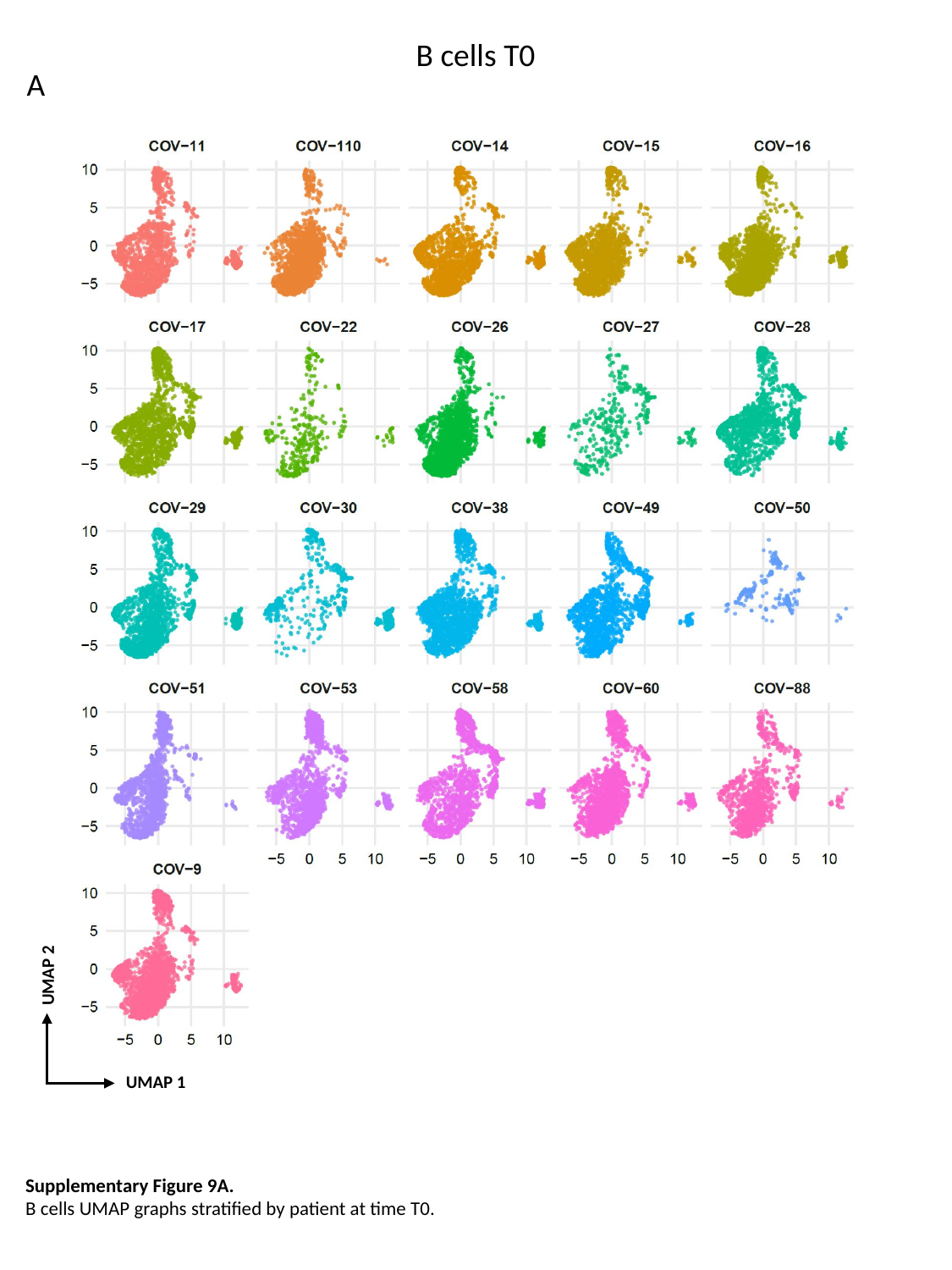

B cells T0
A
UMAP 2
UMAP 1
Supplementary Figure 9A.
B cells UMAP graphs stratified by patient at time T0.

## Slide 17
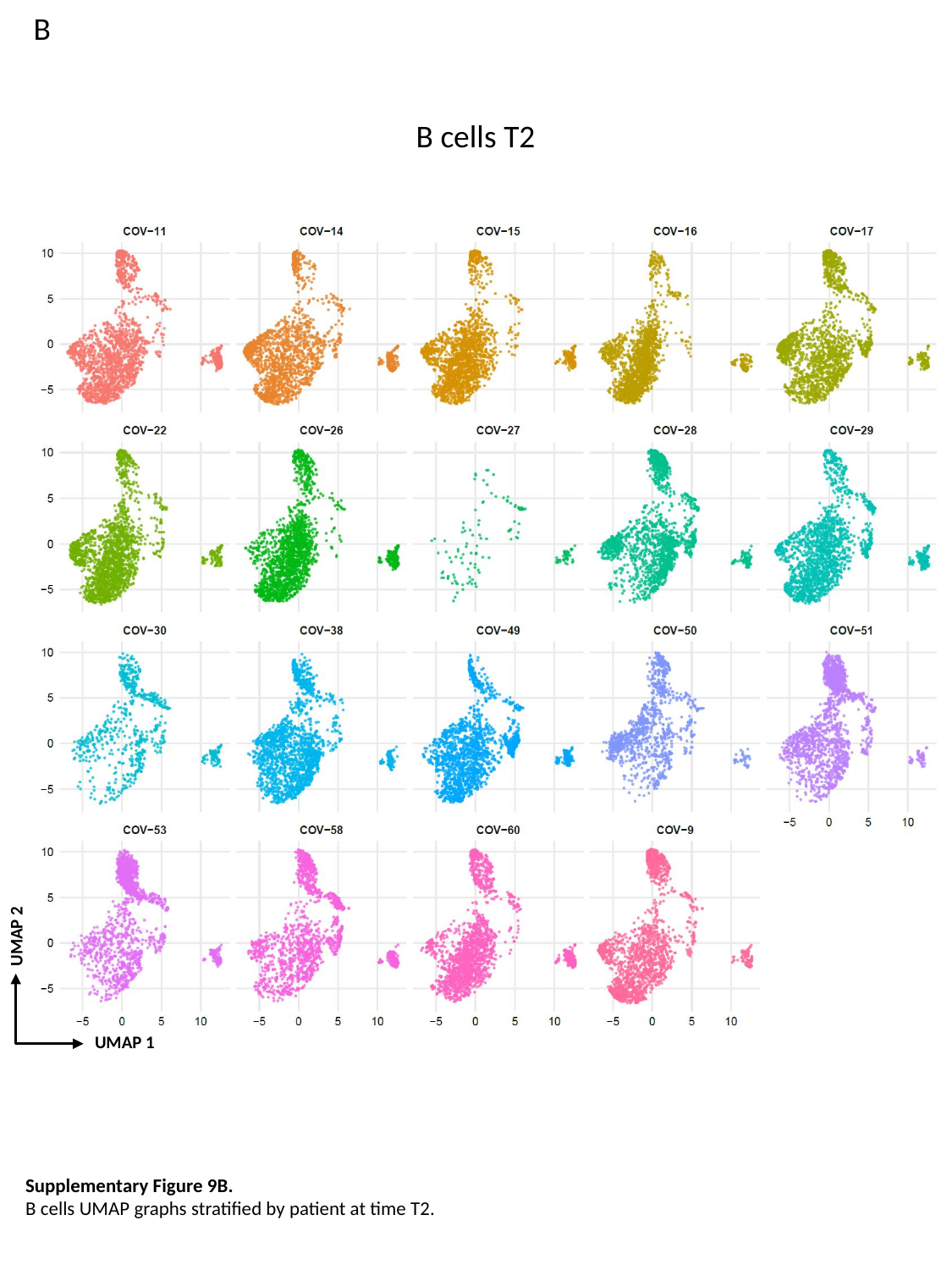

B
B cells T2
UMAP 2
UMAP 1
Supplementary Figure 9B.
B cells UMAP graphs stratified by patient at time T2.

## Slide 18
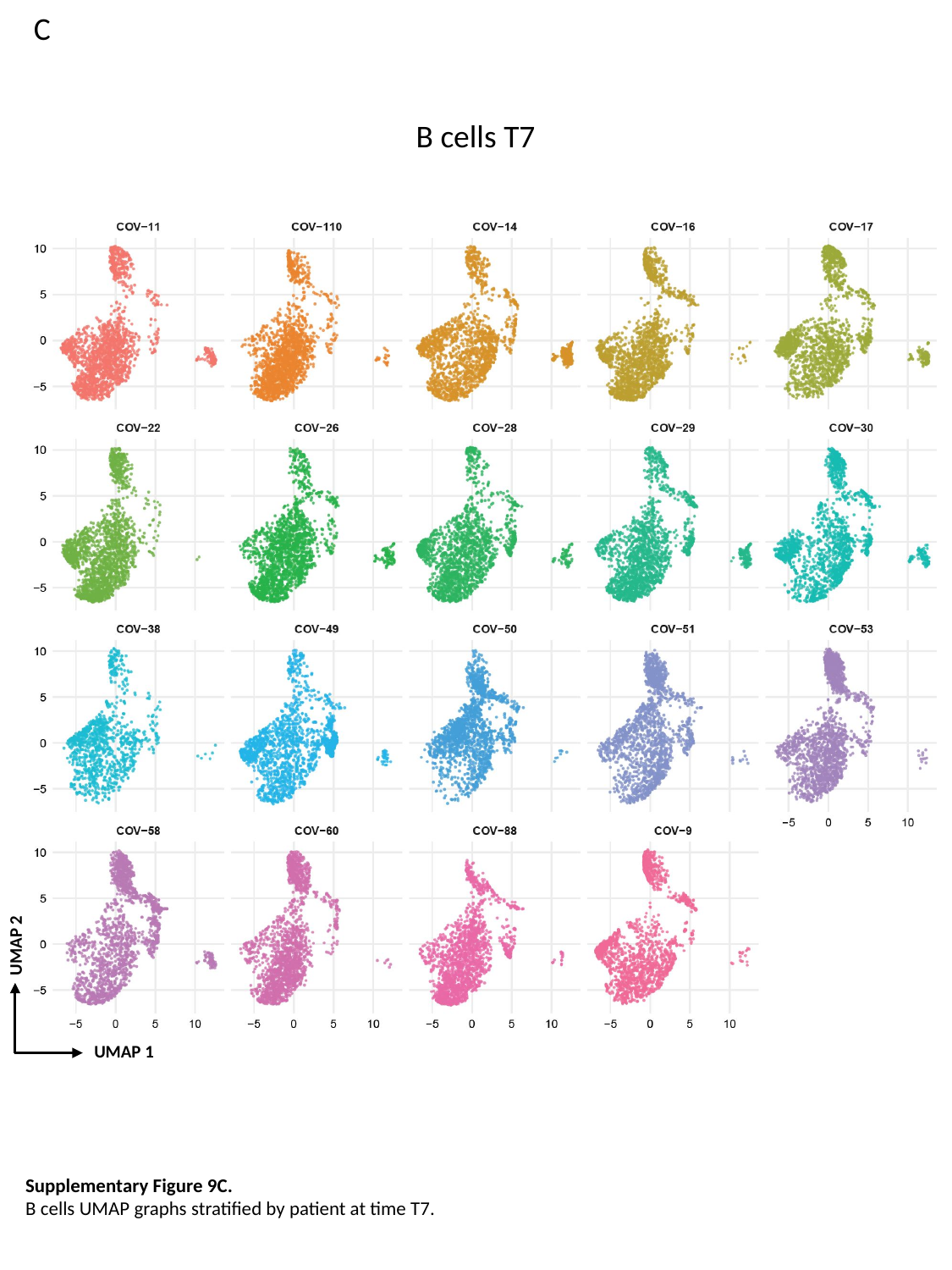

C
B cells T7
UMAP 2
UMAP 1
Supplementary Figure 9C.
B cells UMAP graphs stratified by patient at time T7.

## Slide 19
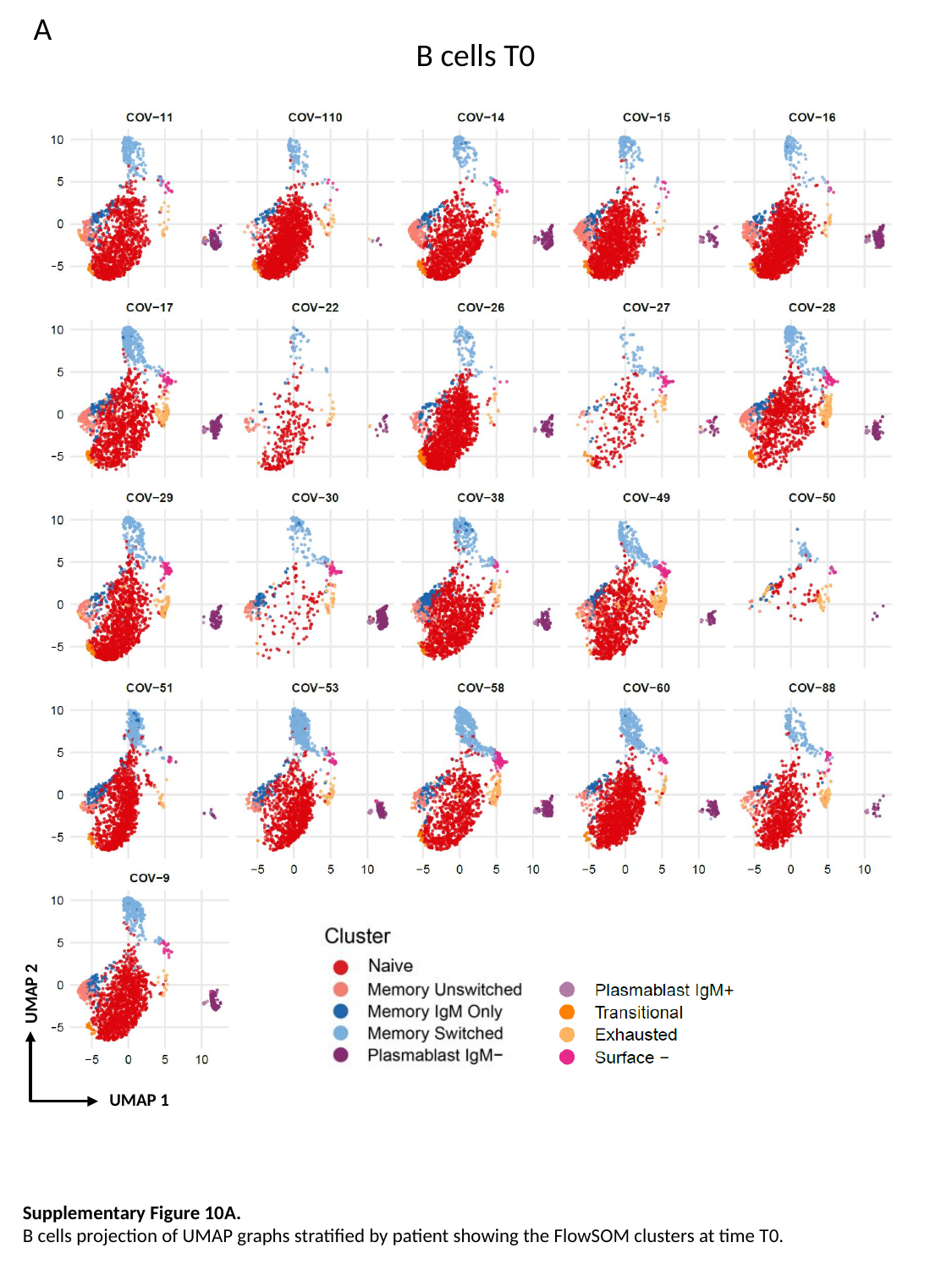

A
B cells T0
UMAP 2
UMAP 1
Supplementary Figure 10A.
B cells projection of UMAP graphs stratified by patient showing the FlowSOM clusters at time T0.

## Slide 20
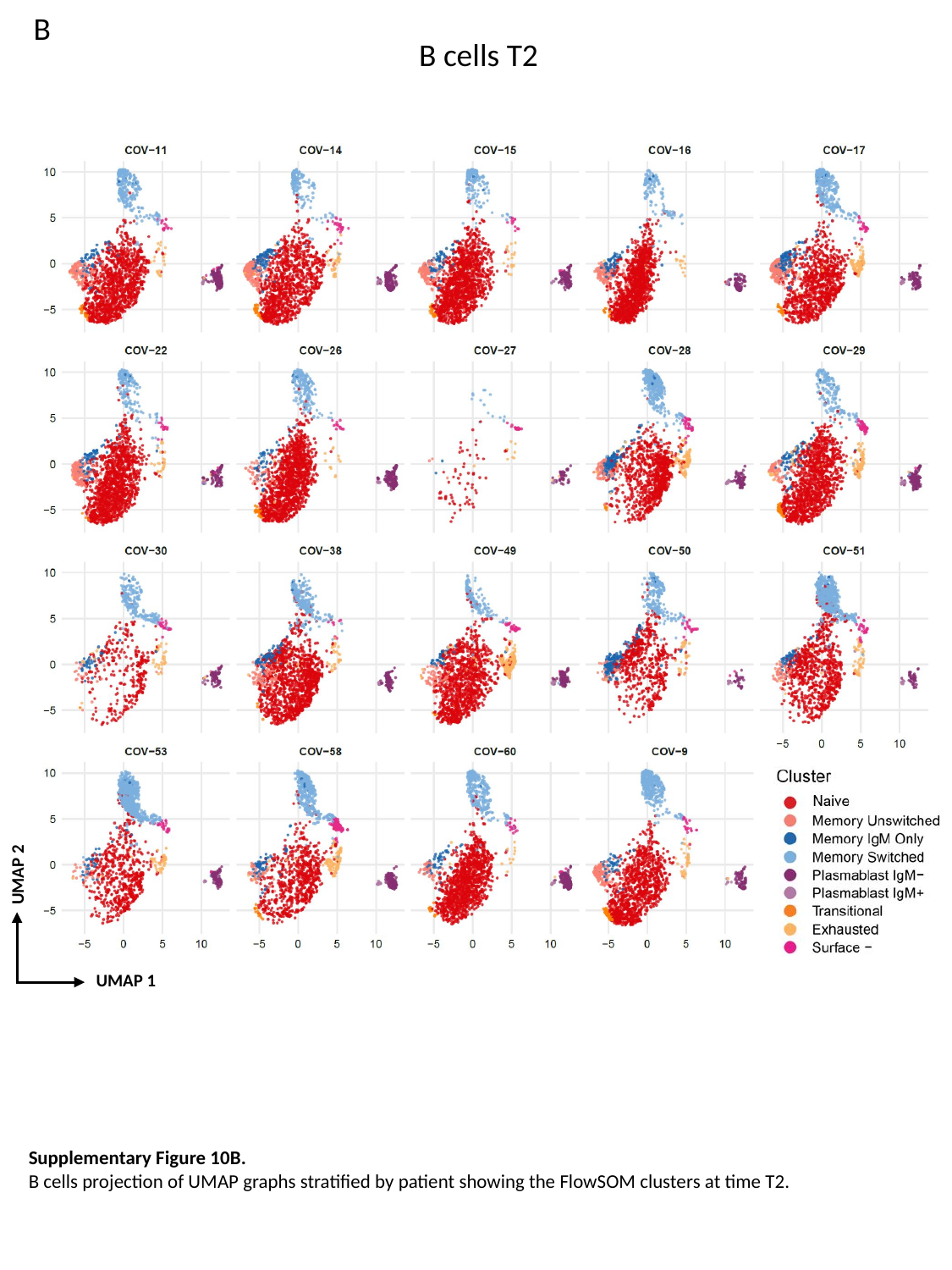

B
B cells T2
UMAP 2
UMAP 1
Supplementary Figure 10B.
B cells projection of UMAP graphs stratified by patient showing the FlowSOM clusters at time T2.

## Slide 21
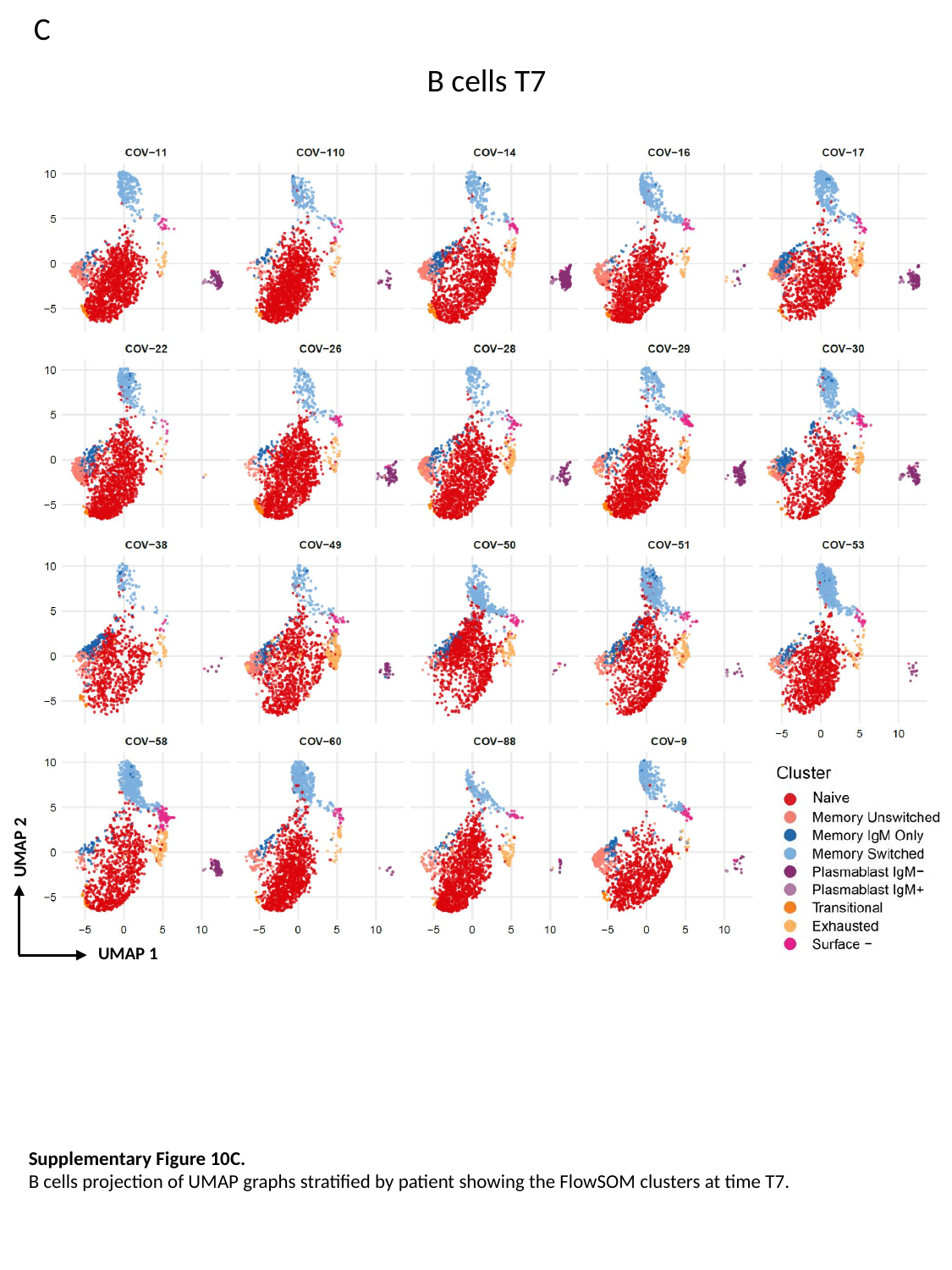

C
B cells T7
UMAP 2
UMAP 1
Supplementary Figure 10C.
B cells projection of UMAP graphs stratified by patient showing the FlowSOM clusters at time T7.

## Slide 22
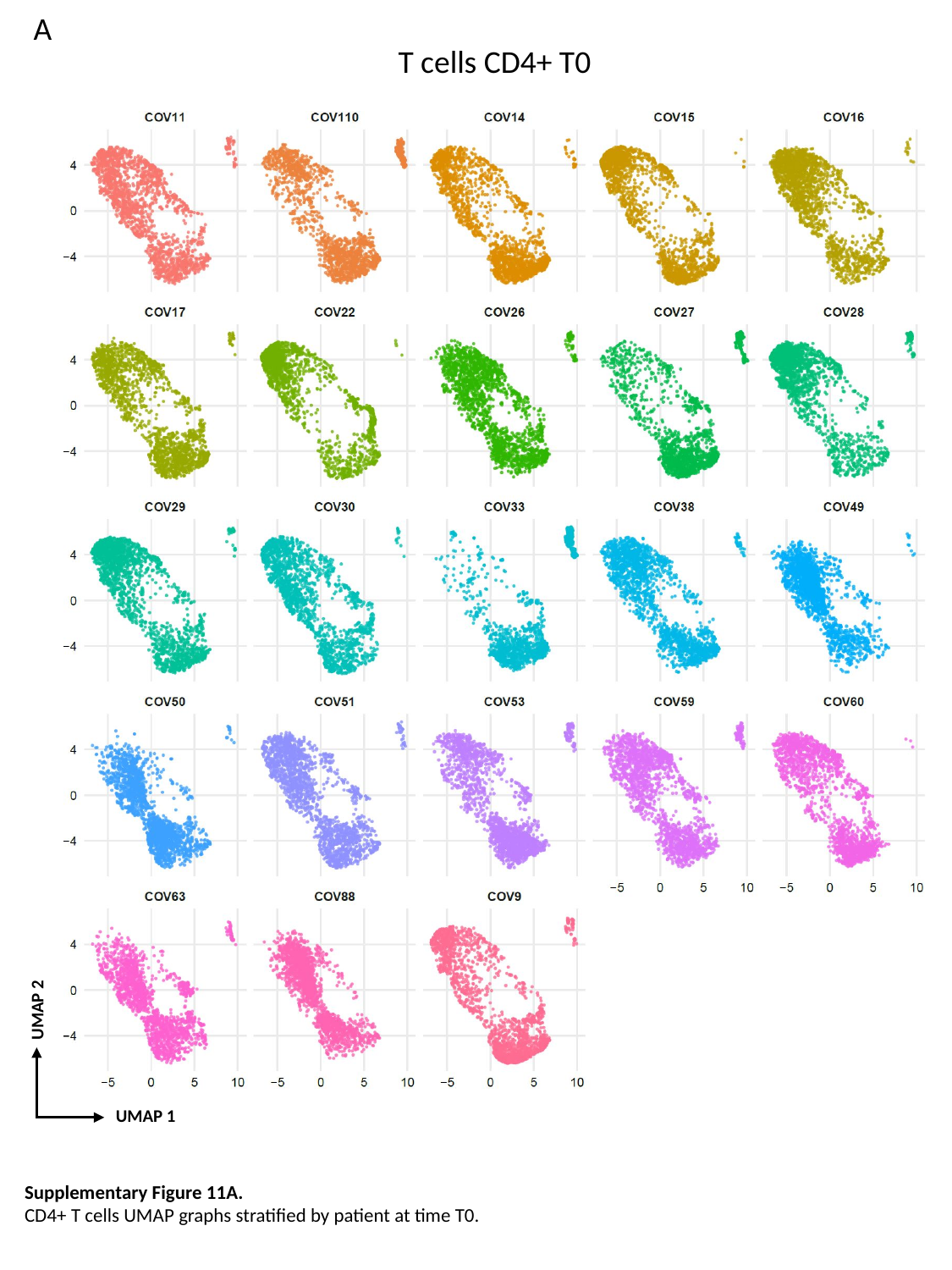

A
T cells CD4+ T0
UMAP 2
UMAP 1
Supplementary Figure 11A.
CD4+ T cells UMAP graphs stratified by patient at time T0.

## Slide 23
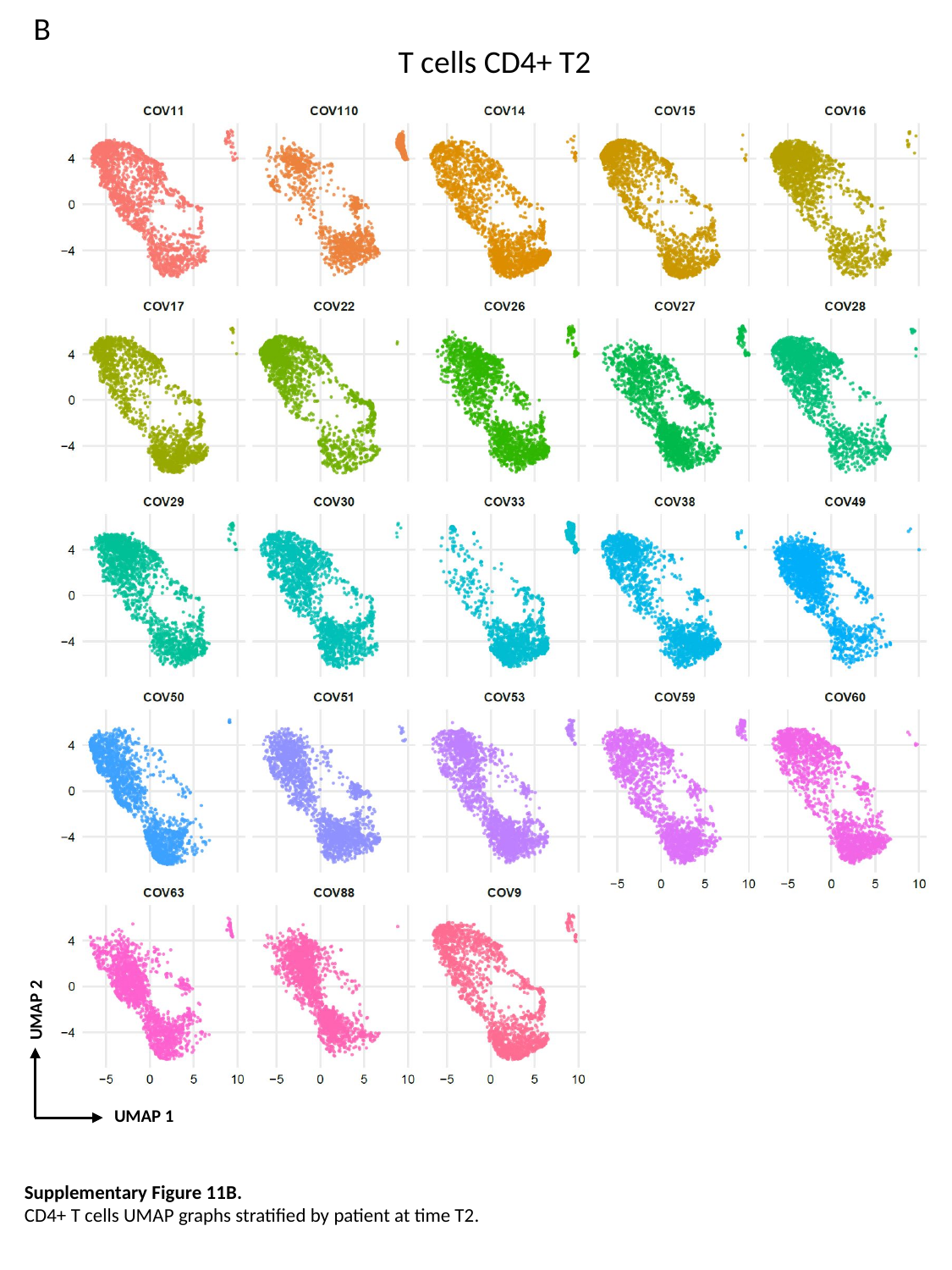

B
T cells CD4+ T2
UMAP 2
UMAP 1
Supplementary Figure 11B.
CD4+ T cells UMAP graphs stratified by patient at time T2.

## Slide 24
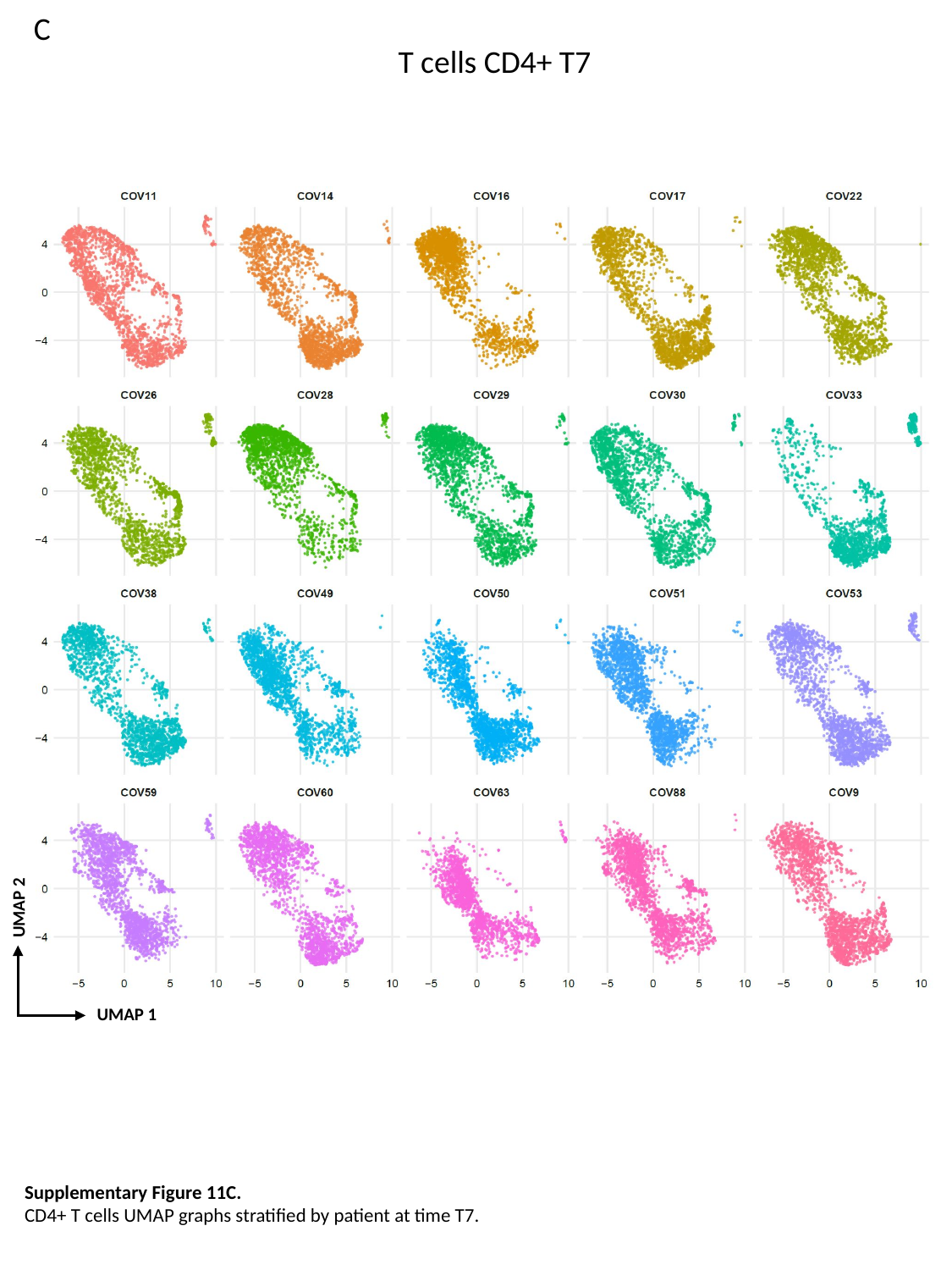

C
T cells CD4+ T7
UMAP 2
UMAP 1
Supplementary Figure 11C.
CD4+ T cells UMAP graphs stratified by patient at time T7.

## Slide 25
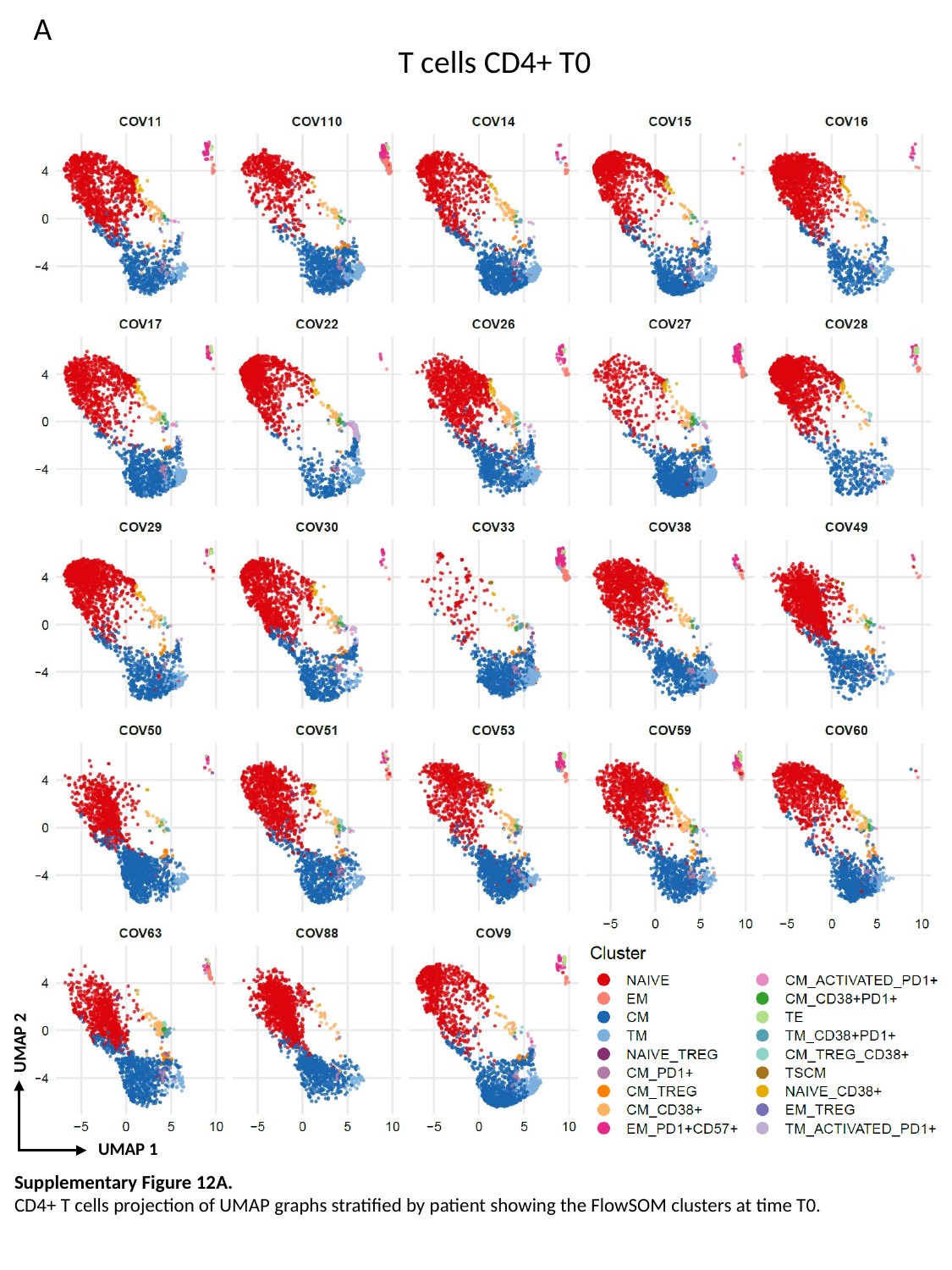

A
T cells CD4+ T0
UMAP 2
UMAP 1
Supplementary Figure 12A.
CD4+ T cells projection of UMAP graphs stratified by patient showing the FlowSOM clusters at time T0.

## Slide 26
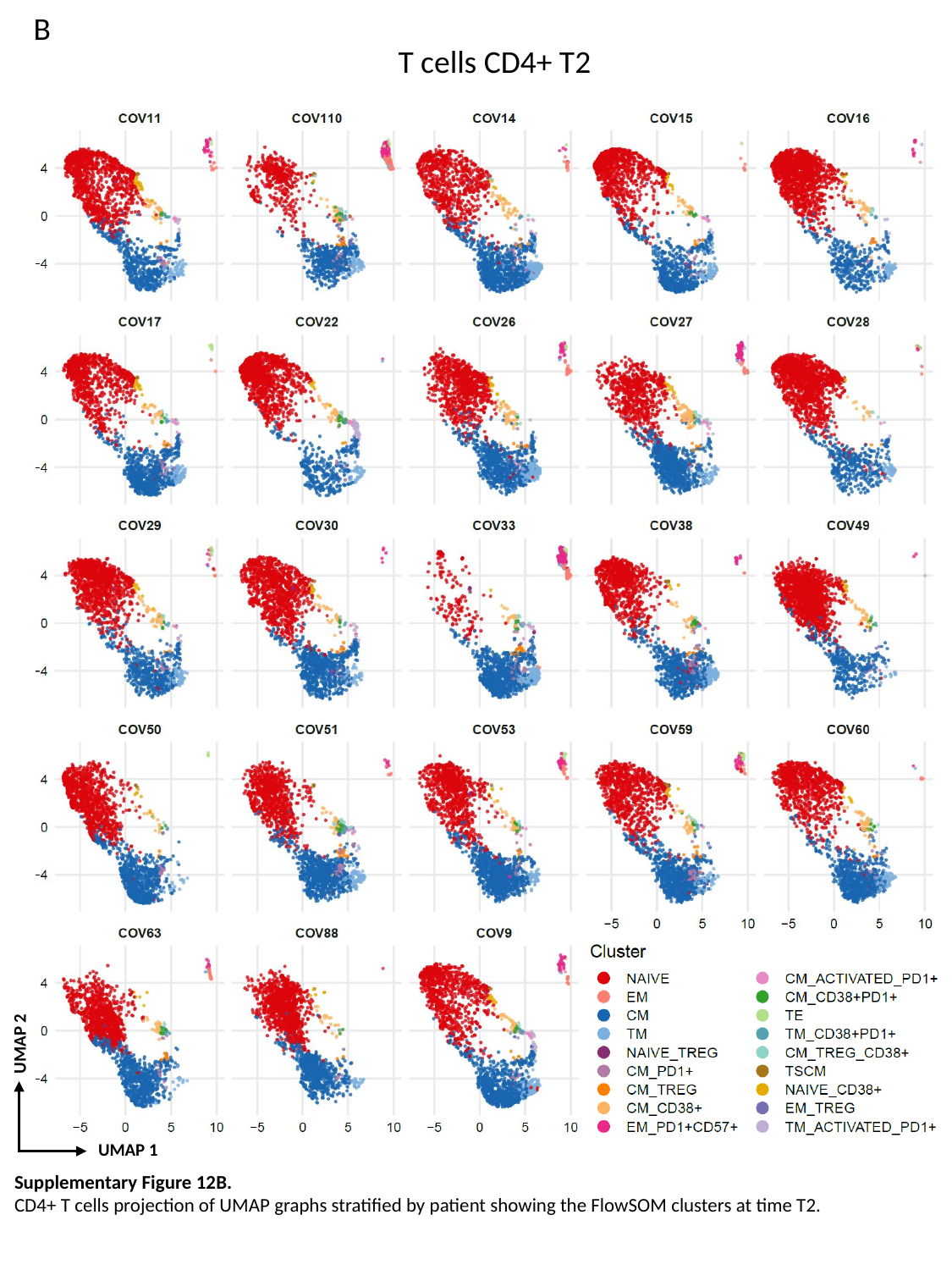

B
T cells CD4+ T2
UMAP 2
UMAP 1
Supplementary Figure 12B.
CD4+ T cells projection of UMAP graphs stratified by patient showing the FlowSOM clusters at time T2.

## Slide 27
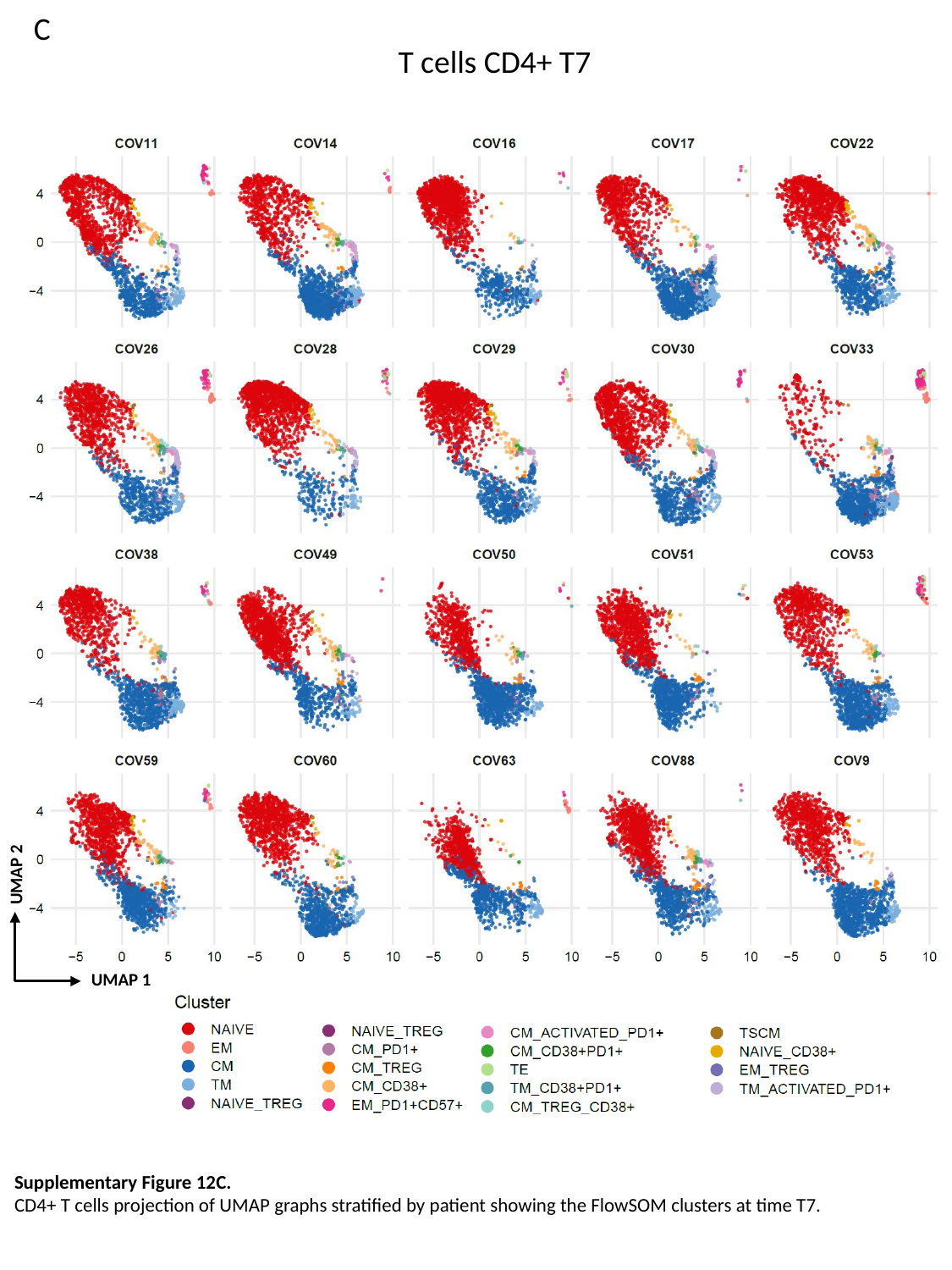

C
T cells CD4+ T7
UMAP 2
UMAP 1
Supplementary Figure 12C.
CD4+ T cells projection of UMAP graphs stratified by patient showing the FlowSOM clusters at time T7.

## Slide 28
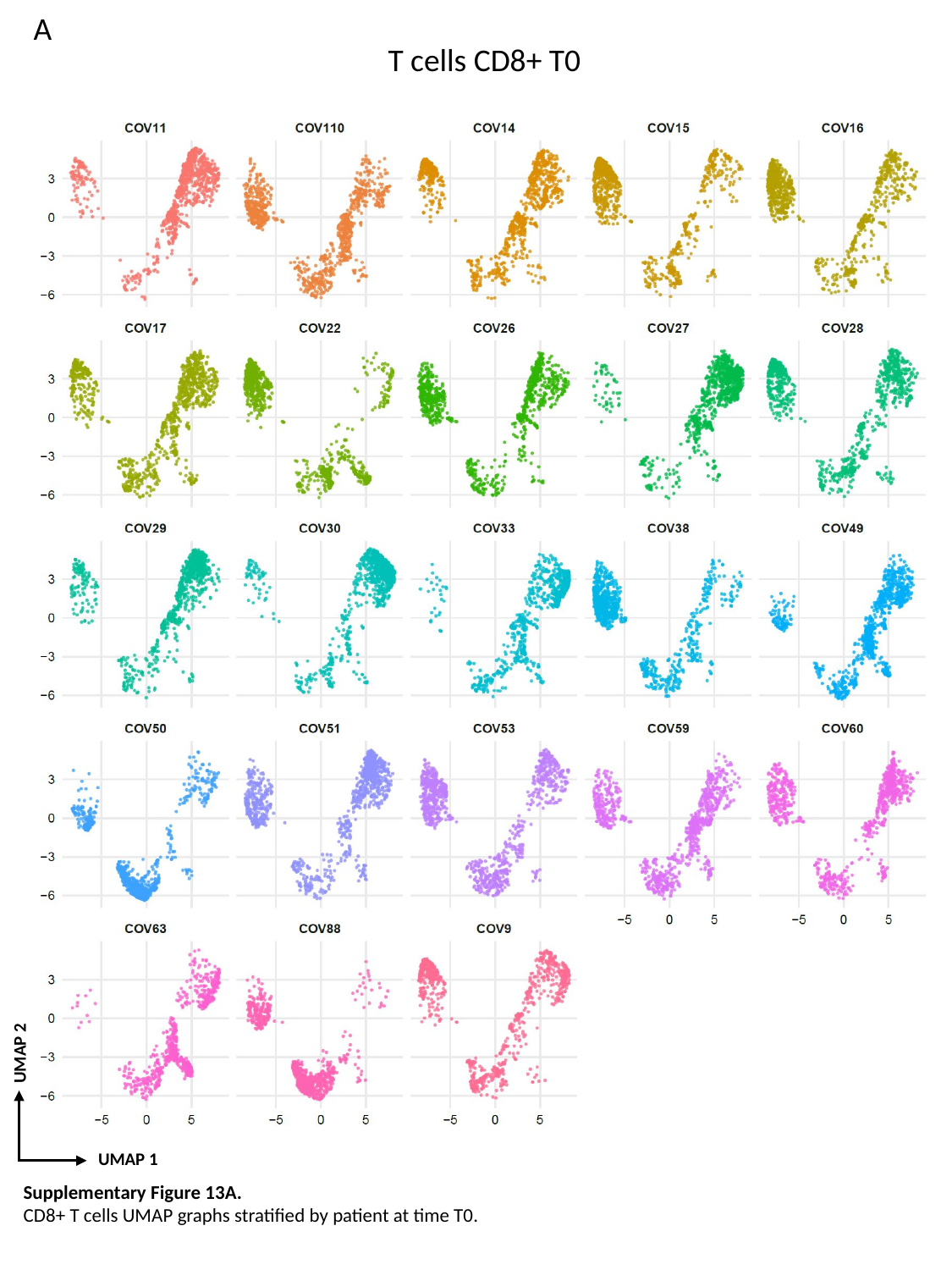

A
T cells CD8+ T0
UMAP 2
UMAP 1
Supplementary Figure 13A.
CD8+ T cells UMAP graphs stratified by patient at time T0.

## Slide 29
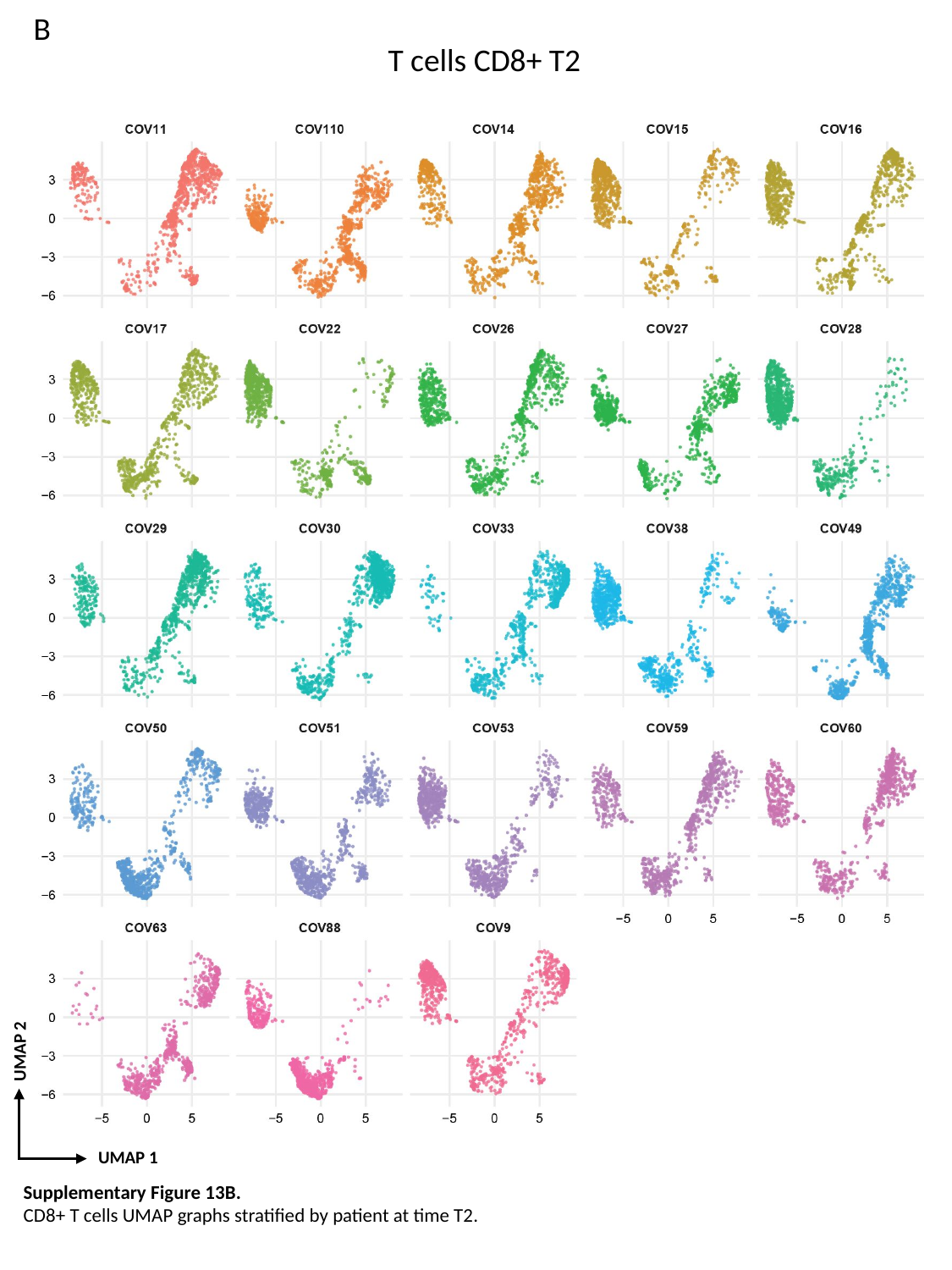

B
T cells CD8+ T2
UMAP 2
UMAP 1
Supplementary Figure 13B.
CD8+ T cells UMAP graphs stratified by patient at time T2.

## Slide 30
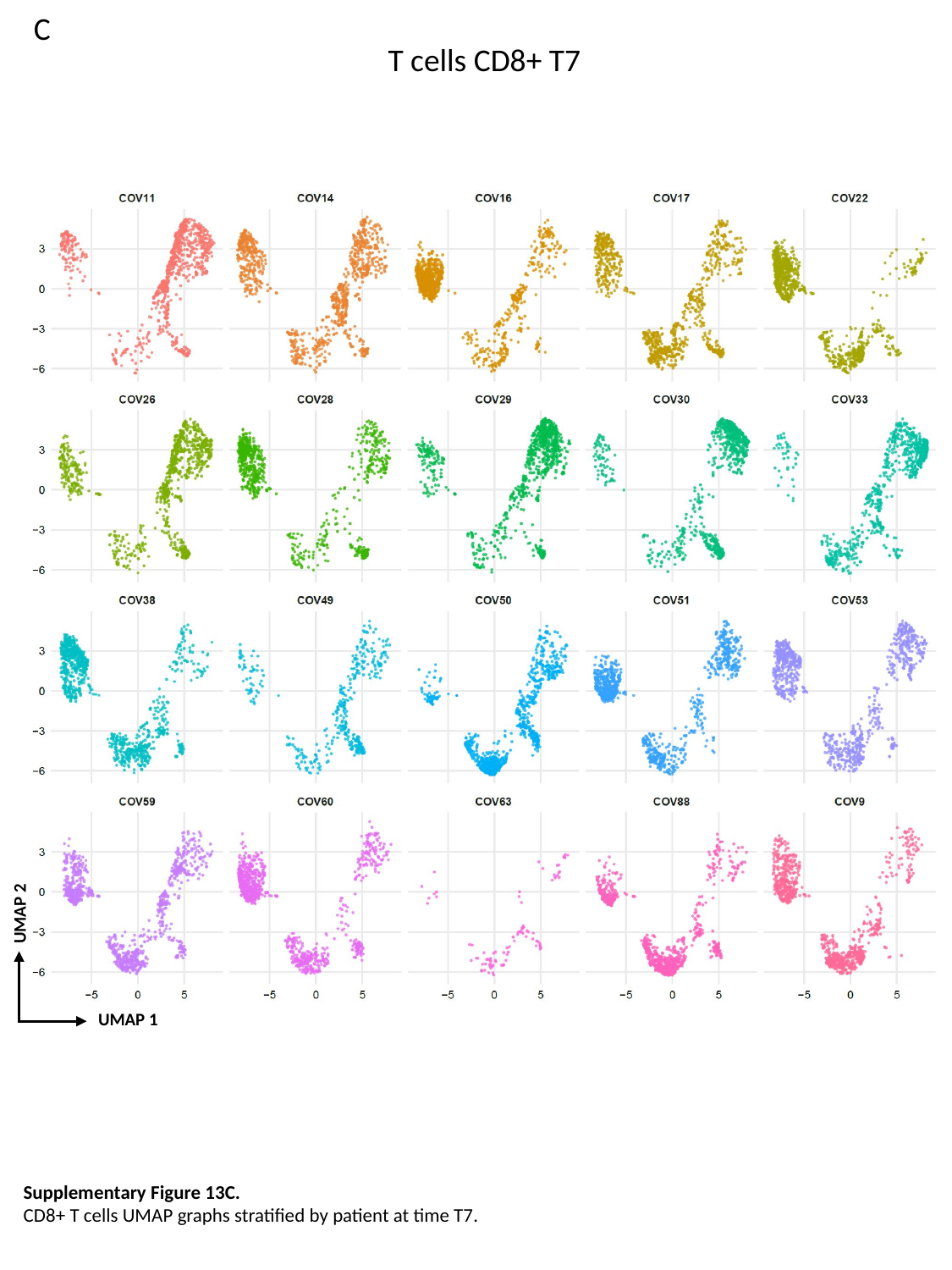

C
T cells CD8+ T7
UMAP 2
UMAP 1
Supplementary Figure 13C.
CD8+ T cells UMAP graphs stratified by patient at time T7.

## Slide 31
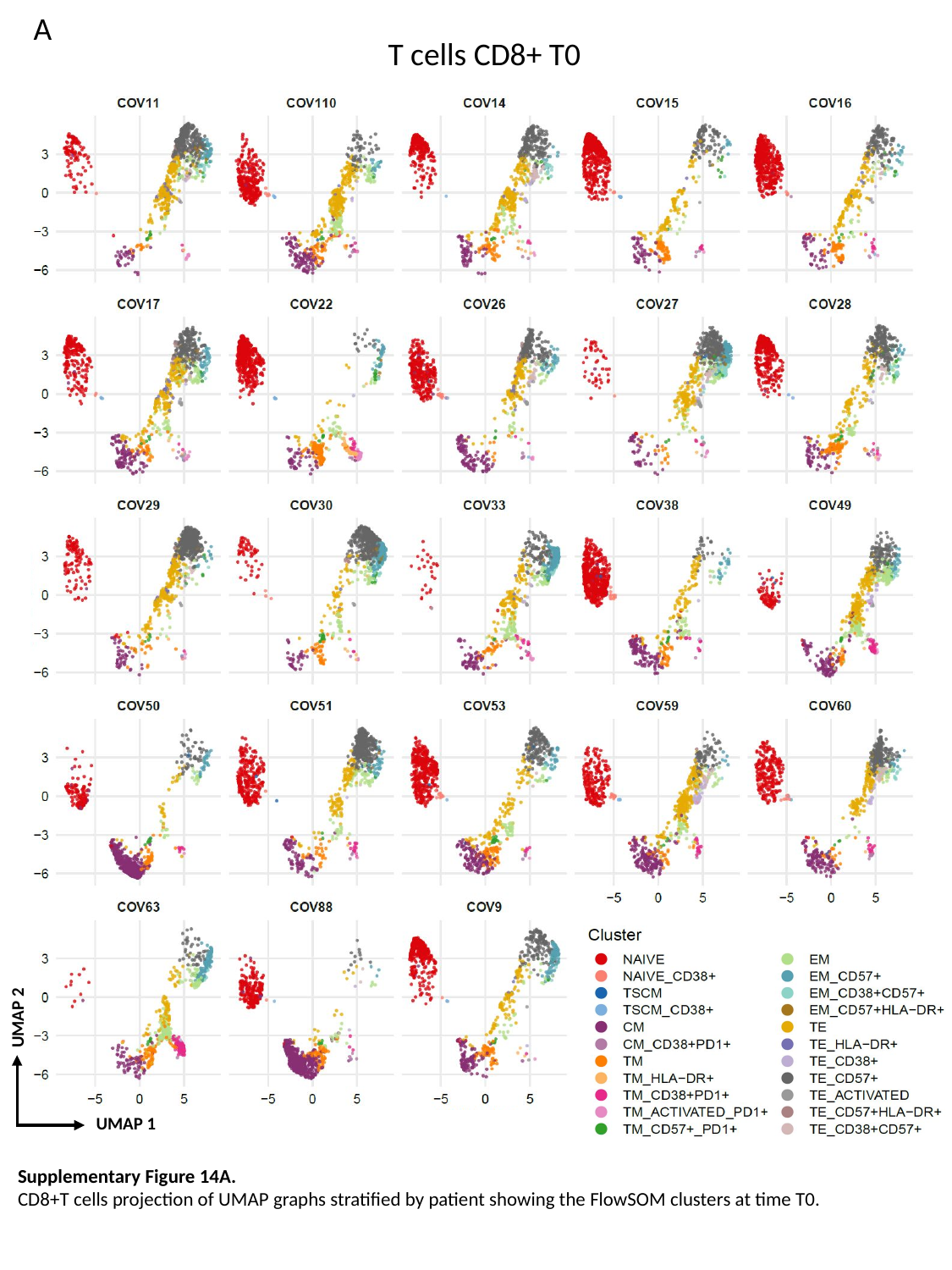

A
T cells CD8+ T0
UMAP 2
UMAP 1
Supplementary Figure 14A.
CD8+T cells projection of UMAP graphs stratified by patient showing the FlowSOM clusters at time T0.

## Slide 32
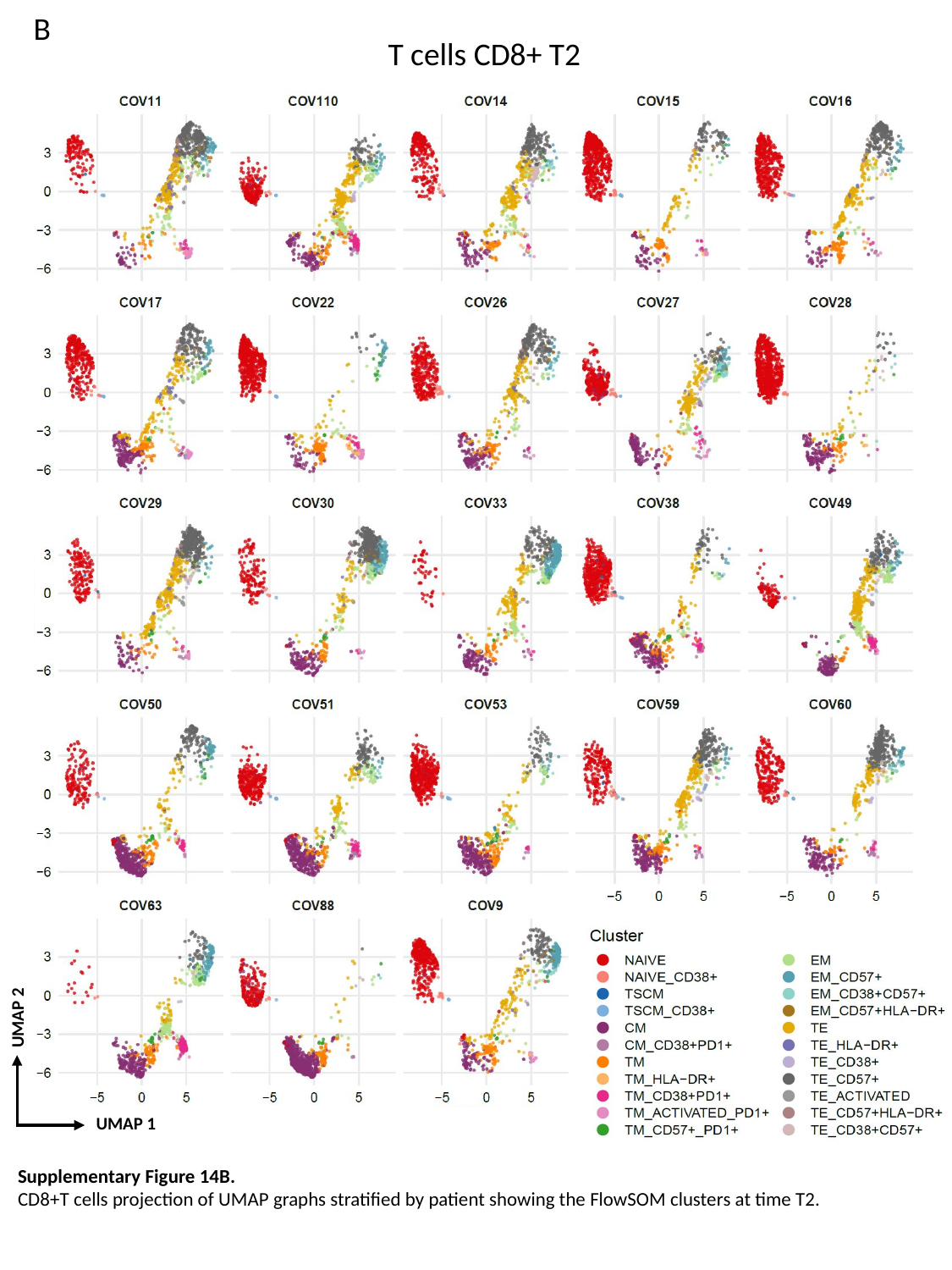

B
T cells CD8+ T2
UMAP 2
UMAP 1
Supplementary Figure 14B.
CD8+T cells projection of UMAP graphs stratified by patient showing the FlowSOM clusters at time T2.

## Slide 33
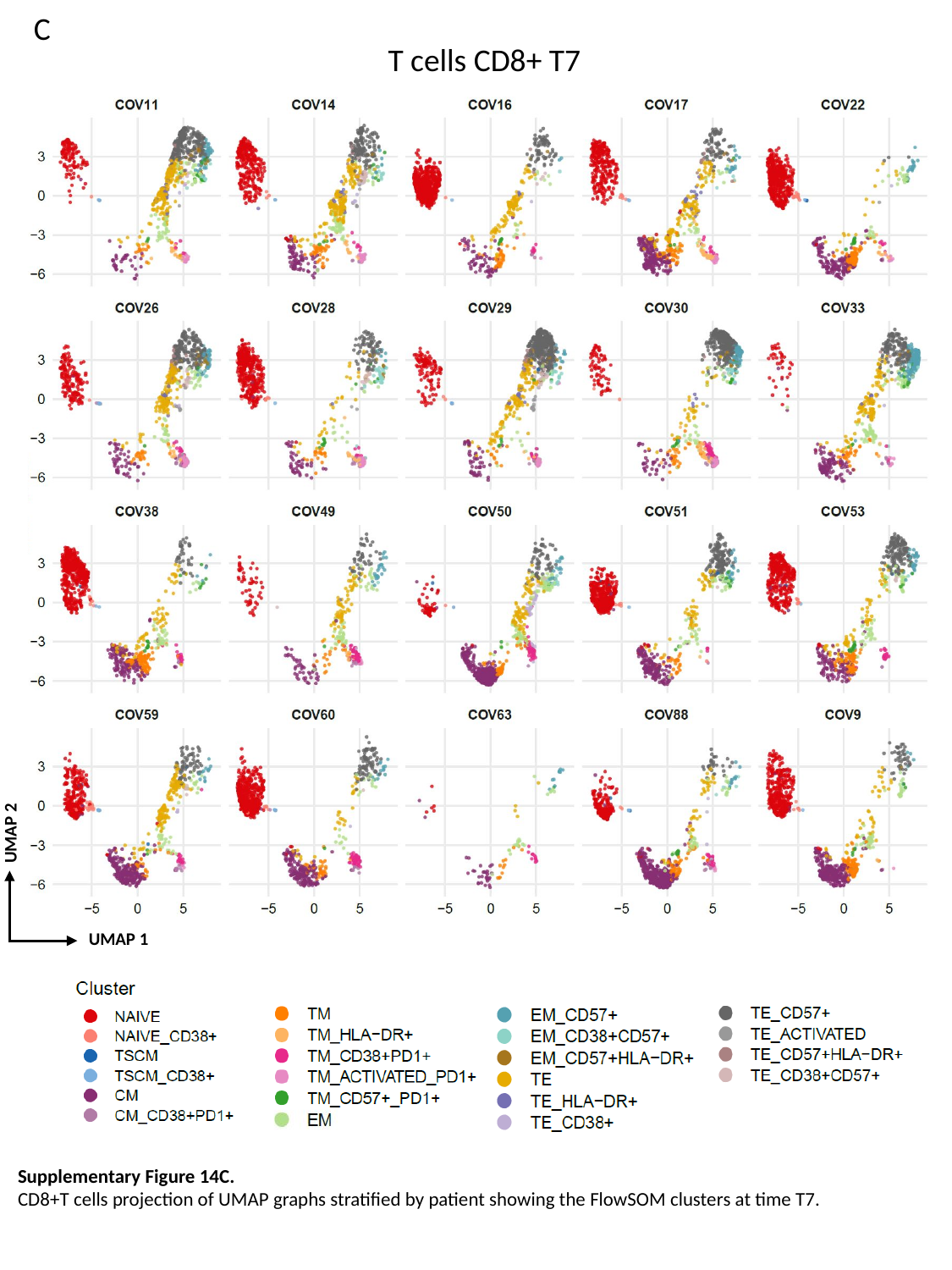

C
T cells CD8+ T7
UMAP 2
UMAP 1
Supplementary Figure 14C.
CD8+T cells projection of UMAP graphs stratified by patient showing the FlowSOM clusters at time T7.
