# Supplementary material for: Prognostic immune markers identifying patients with severe COVID-19 who respond to tocilizumab
Source: Front Immunol. 2023 May 5;14:1123807. doi: 10.3389/fimmu.2023.1123807 (PMC10196248; doi:10.3389/fimmu.2023.1123807)
Supplement: Supplementary file 2 [file Table_1.docx]

**Supplementary Table 1.** Demographic and clinical characteristics of COVID-19 patients responding or not to tocilizumab.

These data are referred to hospital admission, before starting therapy (T0).

| **Variable** | **RESPONDERS**  **(n=13)** | **NON-RESPONDERS**  **(n=10)** |
| --- | --- | --- |
| **Demographic characteristics** |  |  |
| Age (mean years, range) | 60.1 (40 – 85) | 69.9 (53 – 81) |
| Sex (M,%) | 69.2 | 80.0 |
| **Clinical characteristics** |  |  |
| Respiratory rate | 24 (16 – 37) | 29.5 (18 – 40) |
| Heart rate | 77.4 (60 – 112) | 93.6 (83 – 148) |
| SOFA score, mean (range) | 2.5 (1 – 4) | 3.7 (2 – 7) |
| Coexisting conditions |  |  |
| Type 2 diabetes, N (%) | 0 (0) | 2 (20) |
| Cardiovascular Dis., N (%) | 1 (8.3) | 1 (10) |
| Obesity, N (%) | 0 (0) | 1 (10) |
| **Arterial blood gas analysis** |  |  |
| pO_2_, mmHg (range) | 64.3 (47.2 – 96.5) | 59.3 (49.4 – 67.3) |
| sO_2_, % (range) | 90.7 (62.0 – 97.8) | 91.5 (86.6 – 94.6) |
| pCO_2_, mmHg (range) | 37.0 (31.6 – 43.6) | 35.3 (30.0 – 40.1) |
| pO_2_/FiO_2_ (range) | 231.5 (72.0 – 296.0) | 162.1 (52 – 292.0) |
| **Blood parameters** |  |  |
| ALT, U/L (range) | 43.7 (15.0 – 75.0) | 27.8 (19.0 – 40.0) |
| Total bilirubin, mg/dL (range) | 0.7 (0.4 – 1.1) | 0.6 (0.3 – 1.46) |
| CK, U/L (range) | 195.7 (24.0 – 442.0) | 210.2 (52.0 – 505.0) |
| Creatinine, mg/dL (range) | 1.0 (0.7 – 1.3) | 1.6 (0.4 – 6.6) |
| D-dimer, ng/mL (range) | 1,194 (510.0 – 2,140) | 1,481 (650.0 – 3,300) |
| LDH, U/L (range) | 757.5 (340 – 1,307) | 677.3 (526.0 – 846.0) |
| CRP, mg/dL (range) | 13.6 (5.0 – 24.0) | 16.8 (5.0 – 25.0) |

pCO_2_, partial pressure of carbon dioxide; pO_2_, partial pressure of oxygen; sO_2_, oxygen saturation; pO_2_/FiO_2_, fraction of inspired oxygen; ALT, alanine aminotransferase; CK, creatine kinase; LDH, lactate dehydrogenase; CRP, C-reactive protein; NIV, non-invasive ventilation; IMV, invasive mechanical ventilation; Dis, disease.
